# The Lateral Habenula Is Necessary for Maternal Behavior in the Naturally Parturient Primiparous Mouse Dam

**DOI:** 10.1523/ENEURO.0092-24.2024

**Published:** 2025-01-08

**Authors:** Jessie Benedict, Robert H. Cudmore, Diarra Oden, Aleah Spruell, David J. Linden

**Affiliations:** ^1^The Solomon H. Snyder Department of Neuroscience, Johns Hopkins University School of Medicine, Baltimore, Maryland; ^2^Department of Physiology and Membrane Biology, University of California-Davis School of Medicine, Davis, California

**Keywords:** chemogenetics, kainic acid, lateral habenula, linear mixed models, maternal behavior, mouse dam

## Abstract

Mammalian parenting is an unusually demanding commitment. How has the reward system been co-opted to ensure parental care? Previous work has implicated the lateral habenula (LHb), an epithalamic nucleus, as a potential intersection of parenting behavior and reward. Here, we examine the role of the LHb in the maternal behavior of naturally parturient primiparous mouse dams. We show that kainic acid lesions of the LHb induced a severe maternal neglect phenotype in dams toward their biological pups. Next, we demonstrate that chronic chemogenetic inactivation of the LHb using inhibitory DREADDs impaired acquisition and performance of various maternal behaviors, such as pup retrieval and nesting. We present a random intercept model suggesting LHb inactivation prevents the acquisition of pup retrieval, a novel maternal behavior in primiparous mouse dams, and decreases nest building performance, an already-established behavior, in primiparous mouse dams. Lastly, we examine the spatial histology of kainic acid-treated dams with a random intercept model, which suggests the role of LHb in maternal behavior may be preferentially localized at the posterior aspect of this structure. Together, these findings serve to establish the LHb as required for maternal behavior in the mouse dam, thereby complementing previous findings implicating the LHb in parental behavior using pup-sensitized virgin female mice.

## Significance Statement

Work conducted using rats in the 1990s suggested an important role of the lateral habenula (LHb) in maternal behavior, but this area of research has since lain dormant. In the interim, the LHb has been garnering attention as a hub for punishment signaling. Recently, interest in the LHb's role in maternal behavior was renewed, with an important paper examining LHb function during pup-directed behaviors in pup-sensitized virgin female mice. But it is unknown how closely pup-directed behaviors in sensitized virgin females may mimic maternal behavior in natural mouse mothers. This work demonstrates the importance of the LHb in the regulation of natural maternal behavior in the mouse.

## Introduction

Mammalian parenting requires repeated costly choices—limiting foraging time to defend the nest, sharing food, and confronting predators rather than hiding or escaping. Given these costs, the neural circuitry coordinating the shift from self-prioritization to offspring-prioritization should be tightly controlled and robustly rewarding. In addition, maternal behavior is a complex array necessitating specific adjustment across offspring development. For instance, mouse pups old enough to locomote independently no longer require maternal retrieval but instead require new maternal vigilance to ensure pups do not wander dangerously far from the nest. Thus, parenting behavior requires robust reward signaling and flexible decision-making.

Mounting evidence implicates the lateral habenula (LHb), as a neuroanatomical intersection of parenting behavior, flexible decision-making, and reward signaling. LHb lesions prevent the onset of maternal behavior in the rat (Corodimas et al., 1992, 1993; Matthews-Felton et al., 1995; Felton et al., 1998, 1999). The LHb is also required for flexible nonmaternal behaviors, with LHb inactivation causing task-switching and perseverative errors in the rat (Baker et al., 2015, 2017). LHb neurons mediate approach of appetitive stimuli and avoidance of aversive stimuli in the mouse (Stamatakis and Stuber, 2012; Proulx et al., 2014; Stephenson-Jones et al., 2020; Lalive et al., 2022; Mondoloni et al., 2022). The anticipation of punishment and reward omission both cause phasic increases in LHb activity in rhesus macaques (Matsumoto and Hikosaka, 2007). By virtue of its bidirectional connectivity with both the ventral tegmental area (VTA)/substantia nigra pars compacta and the raphe nuclei, the LHb serves as an important integration site for the dopamine and serotonin systems in the brain (Reisine et al., 1982; Amat et al., 2001; L-M. Yang et al., 2008; Kim, 2009; Omelchenko et al., 2009; Jhou et al., 2013; Stamatakis et al., 2013; Amo et al., 2014; Brown et al., 2017). Unsurprisingly, the LHb has been implicated as an important node in the pathophysiology of depression in rodents, rhesus macaques, and humans (Matsumoto and Hikosaka, 2007; Sartorius and Henn, 2007; L-M. Yang et al., 2008; B. Li et al., 2011; Proulx et al., 2014; Shabel et al., 2014; Y. Yang et al., 2018), making the LHb an exciting area of research for peripartum depression and anxiety, which affects 13–19% of human mothers (O’Hara and McCabe, 2013; Dennis et al., 2017; Shovers et al., 2021; Wang et al., 2021).

Recently, interest in the LHb's role in parenting behavior has been renewed (Hu et al., 2020; M. Li, 2022; Mondoloni et al., 2022; Lecca et al., 2023) with an exciting paper by Lecca et al. (2023), examining LHb function during pup-directed behaviors in sensitized virgin female mice. Sensitized virgin female mice are virgin female mice who, after repeated exposure to pups, begin to perform alloparental behaviors. Sensitized virgin females are often used in place of naturally parturient mouse dams, likely to improve experimental throughput. But it remains unclear how the neural dynamics governing pup-directed behaviors in sensitized virgin females compare to those governing maternal behavior in natural dams. Virgin mice learn alloparental care best through social transmission from an experienced dam (Carcea et al., 2021). This is a notably different mechanism than naturally parturient maternal behavior. Though sensitized virgin female mice can perform on par with dams in some pup-directed behaviors, such as latency to retrieve a pup to the nest (Stolzenberg and Rissman, 2011), when motivation is specifically assayed, stark differences between virgins and dams emerge (Gandelman et al., 1970; Salais-López et al., 2021). In a retrieval assay where sensitized virgin female mice and dams could retrieve a live pup or a dead pup from each arm of a T-maze, the sensitized virgin female group retrieved a live pup twice. In the same period, the dam group retrieved dead pups 83 times and live pups 83 times (Gandelman et al., 1970). Thus, sensitized virgin mice are protected from overinvestment in others’ offspring even as dams have undergone such a robust shift to offspring-prioritization that they perform maternal behavior for dead pups.

Here, we have sought to test the hypothesis that the LHb is necessary for maternal behavior by using primiparous mouse dams and their litters to examine the effect of LHb kainic acid lesions and chemogenetic inactivation using designer receptors exclusively activated by designer drugs (DREADDs) on maternal behavior.

**Table 1.** Summary table of all statistical testing performed for the primary figures

Figure	Graph	Data structure	Type of test	*p* Values	Power (95% C.I. of diff)
Figure 1	1*C*, **	Non-normal	Wilcoxon rank sum test with continuity correction	0.0097	2.897925 × 10^−5^, 3.000000 × 10^1^
Figure 1	1*D*, **	Non-normal	Wilcoxon rank sum test with continuity correction	0.0070	2.000038, 29.000010
Figure 1	1*E*, **	Non-normal	Wilcoxon rank sum test with continuity correction	0.0047	0.9999726, 3.9999092
Figure 2	2*A*, *	Borderline normal/non-normal distribution	Wilcoxon rank sum test with continuity correction	0.0303	229, 6159
Figure 2	2*B*, *	Normal distribution	Welch's two-sample *t* test	0.0213	0.7899646, 6.8571687
Figure 2	2*C*, N.S.	Normal distribution	Welch's two-sample *t* test	0.0868	−26.65959, 326.12626
Figure 2	2*D*	Non-normal, continuous variables	Pearson’s correlation	7.84 × 10^−6^	0.8128328 0.9871671
Figure 3	3*B*; N.S., N.S., *, ***	Non-normal distribution	Wilcoxon rank sum test with continuity correction	0.5286	−5.106843 × 10^−5^, 1.000069 × 10
				0.0623	−8.949650 × 10^−6^, 1.099993 × 10^1^
				0.0305	4.107240 × 10^−5^, 1.400002 × 10^1^
				0.0002	7.000012, 18.999943
Figure 3	3*C*; N.S., ***	Non-normal distribution	Wilcoxon rank sum test with continuity correction	0.4017	−3.580000 × 10^2^, 4.756916 × 10^−5^
				0.0816	−7.670000 × 10^2^, 8.081876 × 10^−6^
				0.0501	−9.28000 × 10^2^, 5.75822 × 10^−5^
				0.0008	−1,152, −449
Figure 4	4*A*; *,**,*,*	Non-normal distribution	Wilcoxon rank sum test with continuity correction	0.0343	3.941284 × 10^−5^, 1.600001 × 10^1^
				0.0083	2.000005, 15.000058
				0.0318	0.9999839, 14.0000286
				0.0439	4.104741 × 10^−5^, 1.299998 × 10^1^
Figure 4	4*B*, N.S., *, N.S., N.S.	Non-normal distribution	Wilcoxon rank sum test with continuity correction	0.0752	−1.806474 × 10^−5^, 2.999900 × 10
				0.0137	2.646538 × 10^−5^, 2.000050 × 10
				0.0984	−1.059384 × 10^−5^, 2.999952 × 10
				0.2892	−0.999988, 2.999952
Figure 5	5*B*	Non-normal distribution	Multivariate multilevel random intercept model	0.2613	3.6798, 6.3702;
				0.0002	−5.9602, −1.9698
Figure 5	5*D*	Non-normal distribution	Multivariate multilevel random intercept model	0.0084	−0.6907868, 1.540787
				0.5760	−1.1799793, 2.129979
Figure 6	6*A*, *	Normal distribution	Welch's two-sample *t* test	0.0400	110.8113, 4159.2554
Figure 6	6*B*	Borderline normal/non-normal	Wilcoxon rank sum test with continuity correction	0.0169	1.013, 3.964
Figure 6	6*C*, N.S.	Normal distribution	Welch's two-sample *t* test	0.1842	−372.56343, 76.67809
Figure 7	7*B*	Non-normal distribution	Multivariate multilevel random intercept model	0.178	6.209012, 8.697655
				0.0000	−8.697655, −6.209012
				0.0000	−7.281687, −3.346798
Figure 7	7*D*	Non-normal distribution	Multivariate multilevel random intercept model	0.1903	5.774927, 7.757498
				0.0000	−7.757498, −5.774927
				0.0000	−8.625390, −5.490670

## Materials and Methods

### Experimental animals

Pup-naive virgin female C57BL/6NCrl mice (Charles River Laboratories stock #027; RRID:IMSR_CRL:027) aged 6–9 weeks were randomly assigned to a treatment group for all experiments. Male C57BL/6NCrl mice (Charles River Laboratories stock #027; RRID:IMSR_CRL:027) were used for breeding purposes only, as all experiments herein examine maternal behavior. All animal procedures were performed in accordance with the Johns Hopkins University Animal Care and Use Committee's regulations. Animals were housed on a 12 h:12 h standard light/dark cycle. Food and water were available *ad libitum*.

### Kainic acid lesion experiments

#### Stereotaxic injection surgery

Mice were anesthetized using vaporized isoflurane (5% for induction, 1.5% for maintenance) and placed in a stereotactic frame (Leica, Angle 2). Ophthalmic ointment was applied and rectal temperature and respiration rate were monitored throughout surgery. The mice were shaved, and iodine was swabbed on the scalp. Mice were given lidocaine (0.5% lidocaine, 0.02 ml) and dexamethasone (0.20 ml at 0.4 mg/ml) subcutaneously under the scalp. Mice received diazepam (2.5 mg/kg, i.p.) as an analgesic and an anticonvulsant and enrofloxacin (2.5 mg/kg i.p.) as an injectable antibacterial solution. An incision was made on the scalp to reveal the skull surface. The bregma and lambda were aligned in the mediolateral (ML) and dorsoventral (DV) planes within 30 μm of each other, and the points 2 mm lateral to the bregma in each hemisphere were aligned within 30 μm of each other in both the anteroposterior (AP) and DV planes. A craniotomy was performed using a rotary tool affixed to the stereotactic frame. The drill rotated a 0.9 mm drill burr which was irrigated with ice-cold saline. Injections were performed with a Nanoject II (Drummond) using a glass pipette with a tip diameter of 12.5–22.5 μm. Depending on experimental group assignment, either sterile saline (0.9% sodium chloride, Hospira) or kainic acid (1 mg/ml dissolved in sterile saline, Tocris Bioscience; catalog #0222) was injected into the LHb. Injections of 101.2 nl volume were made bilaterally at −1.50 AP, ±0.42 ML, and −2.70 DV. Approximately 5 min elapsed before retracting the pipette from the brain. During the retraction, slight negative pressure was applied to the pipette through the Nanoject II. The scalp was sutured with absorbable polyglycolic acid suture, and iodine was reapplied. Mice were then given IP buprenex (0.05 mg/kg) and 0.5 ml intraperitoneal sterile saline to prevent dehydration and placed under a heat lamp for observation. Once a mouse awoke from anesthesia and was ambulating normally, it was returned to a shared cage. Mice were supplied with hydration gel postoperatively and their sutures were monitored daily for 7 d.

#### Breeding and pregnancy

Animals were given at least 1 week to recover postoperatively and were then housed in a harem breeding scheme with 2–3 females per breeding male. Mice remained group housed until approximately Embryonic Day (E)17 when pregnant females were moved to cages within individual behavior boxes to allow for continuous video monitoring as they neared parturition.

#### Naturalistic maternal behavior tests

Lactation Day 0 (LD0) was demarcated as the first day live pups were seen in the cage by 1200 h. Naturalistic maternal behavior tests (MBTs) were conducted on LD1. Standard MBTs were planned to start on LD2, but these were never performed since nearly all the kainic acid-treated dams’ litters had died of neglect by LD2. The naturalistic MBTs were scored from 30 min of video in which all dams demonstrated activity, between 1400 and 1600 h.

#### Behavior scoring

The 30 min naturalistic MBT video was scored blind to experimental group, using custom software detailed in Benedict and Cudmore (2023) consistent with standard MBTs (Numan and Callahan, 1980; Matthews-Felton et al., 1995; Felton et al., 1998; Nephew and Bridges, 2011; Wu et al., 2014; Benedict and Cudmore, 2023). Thirty 10 s video windows were randomly selected from each assay and scored in a randomized order. Each window was scored for the presence or absence of the following behavioral states: all pups gathered, all pups nested, arched-back hovering/visible nursing, time with pups, pup interactions (retrieval, inspection, anogenital licking), nest building, solo activity, solo rest, pup lacking visible milk spot, and dead pup visible. Additionally, a blinded experimenter scored each nest at the end of the MBT. Nests were scored using integers 0–4 as follows: 0, no nest is attempted; 1, poor nest, not all of the nesting material was used, and it lacks structure; 2, fair nest, all the nesting material was used, but the nest lacks structured walls; 3, good nest, all nesting material was used, and the nest has low walls; and 4, excellent nest, all the nesting material was used, and the nest has high structured walls (Numan and Callahan, 1980). Scores for all pups nested were not reported in Extended Data Figure 1-1 since all values were identical to those reported for all pups gathered.

### Chronic chemogenetic inactivation with DREADDS

#### Stereotaxic injection surgery

All procedures were identical to those already described for Kainic acid lesion experiments—Stereotaxic injection surgery, except for the following differences: mice received preoperative buprenex (1 mg/kg) rather than diazepam. The drill burr for the craniotomy was 1.2 mm rather than 0.9 mm, to enable multiple injection sites. Rather than kainic acid or saline, depending on group assignment, either experimental AAV2-hSyn-hM4D(Gi)-mCherry (RRID: Addgene_50475) or control AAV2-hSyn-mCherry virus (RRID: Addgene_114472) was injected into the LHb. Injections of 101.2 nl were made bilaterally at −1.45 AP and −1.55 AP, ±0.42 ML, and −2.70 DV or, later, at −1.28 AP and −1.38 AP and −1.48 AP, ±0.42 ML, and −2.70 DV, as immunohistochemical results from early cohorts showed sparser mCherry expression in the anterior-most aspect of the LHb and so coordinates were adjusted.

#### Breeding and pregnancy

Procedures were identical to those already described for Kainic acid lesion experiments—Breeding and pregnancy, except for the addition of Agonist-21 administration. Approximately 24 h before parturition (range, 6–40 h), each mouse began receiving subcutaneous injections of the hM4Di agonist called Agonist-21 (Hello Bio; dose, 3 mg/kg, HB4888) every 6 h (0900, 1500, 2100, 0300 h) to maintain putative neural inactivation in the LHb (Thompson et al., 2018; Jendryka et al., 2019; Ferrari et al., 2022). Once at least 6 h had elapsed since parturition, the uterus was palpated, and if no additional pups were suspected, injections were shifted to the intraperitoneal route to minimize discomfort. Mice were weighed at 0900 h to determine their dose for that day.

#### Standard MBTs

LD0 was demarcated as the first day live pups were seen in the cage by 1200 h. Behavior experiments were conducted on LD1, LD2, LD3, and LD4, during the first hour of the light cycle. Following Agonist-21 administration, dams were placed in a clean separation chamber for 30 min. While the agonist took effect, the cage was cleaned, food and water were removed for the duration of the assay, and new nesting material was supplied. Pups were examined for the presence or absence of milk spots, signs of physical injury, and weight gain. Just before the 30 min mark, pups were returned to the clean cage and scattered in the three corners opposite a new nestlet. The video recording began at the dam's moment of return to the cage, which commenced the 30 min MBT.

#### Behavior scoring

The standard MBT differs from the naturalistic MBTs (see above, Kainic acid lesion experiments) in that it includes a separation period before the dam is reintroduced to scattered pups. Therefore, the behavior scoring for standard MBTs includes scores for delay to initiate first pup retrieval and delay to complete pup retrieval (Extended Data Fig. 3-1). Since the assay duration was 1,800 s (30 min), animals that failed to initiate or complete pup retrieval by that point received a score of 1,800 s. The standard MBT scores for solo rest, pup lacking visible milk spot, and dead pup visible are not included in Extended Data Figure 3-1 since all values for both groups were zero.

### All experiments

#### Perfusion and tissue processing

On LD4, dams were briefly anesthetized with isoflurane and injected with a ketamine/xylazine cocktail (4 ml/kg) to achieve deep anesthesia. When unresponsive to a toe pinch, the dams were then myocardially perfused with ice-cold 1× PBS and ice-cold 4% PFA (Electron Microscopy Sciences, 20% solution, EM Grade #15713, diluted to 4% in PBS). Brains were postfixed overnight in 4% PFA and then placed in 15% sucrose until they sunk. The same procedure was then repeated using a 30% sucrose solution. The brains were sectioned coronally on a freezing microtome. Forty-micron-thick sections were collected, and alternate sections were then used for immunohistochemistry and histology analysis.

#### Immunohistochemistry

Sections were washed three times in wash buffer (1× PBS + 0.3% Triton X-100 in a 24-well plate) for at least 5 min per wash. Next, they were placed in blocking serum [wash buffer + 5% normal goat serum (Jackson ImmunoResearch Laboratories, RRID: AB_2336990] for 1 h at room temperature or overnight at 4°C. Primary antibodies, anti-NeuN (Millipore Sigma, catalog #MAB377, RRID: AB_2298772) and anti-GPR151 (Sigma-Aldrich, catalog #SAB4500418, RRID: AB_10743815), were added to fresh blocking serum, and sections were incubated for 1–2 h at room temperature or overnight at 4°C. After this step, sections were washed three times in wash buffer for at least 5 min per wash. Sections were then incubated with secondary antibodies Alexa Fluor 488-AffiniPure Goat Anti-Mouse IgG (H + L; Jackson ImmunoResearch Laboratories, catalog #115-545-003, RRID:AB_2338840) and Alexa Fluor 647-AffiniPure Goat Anti-Rabbit IgG (H + L; min X Hu, Ms, Rat Sr Prot) (Jackson ImmunoResearch Laboratories, catalog #111-605-144, RRID: AB 2338078) in fresh blocking serum for 2–4 h at room temperature or overnight at 4°C. Lastly, sections were washed three times in wash buffer for at least 5 min per wash before being mounted with ProLong Diamond AntiFade Mountant with DAPI and allowed to dry overnight.

#### Microscopy and histological analysis

All microscopy and cell counting was conducted blinded to treatment group using a Zeiss 800 inverted confocal microscope. Tiled *z*-stacks were collected with a 20× objective to capture the entire LHb, with 8–11 images analyzed per animal. Image files were stitched using the ZEN Blue software (RRID: SCR_013672) and then exported for cell and volume quantifications. Imaris software (version 9.8, RRID: SCR_007370) was used to outline the LHb across all *z*-slices of a given image, guided by a combination of NeuN and anti-GPR151 immunostaining. Then, all cells within outlined LHb borders were exhaustively counted, using NeuN immunofluorescence for counting neurons in the kainic acid lesion experiments and mCherry immunofluorescence/c-Fos immunofluoescence for counting transgene-expressing cells and c-Fos–expressing cells in the chronic chemogenetic inactivation experiments. Separate blinded scoring was performed of off-target kainic acid lesioning in the kainic acid lesion experiments and transgene expression in the chemogenetic inactivation experiments. For kainic acid off-target lesion scoring, the scoring was conducted semiquantitatively by a blinded scorer. The scorer was first trained on four injection-naive brains stained using the same protocol as the saline and kainic acid group brains. The scorer was trained to assess cell density in the five most relevant neighboring brain regions: the hippocampus, the medial habenula, the paraventricular nucleus of the thalamus, the central lateral nucleus of the thalamus, and the anterior pretectal nucleus. The scale was 0 (no apparent cell loss), 1 (some minor cell loss), 2 (significant cell loss), and 3 (major cell loss). All kainic acid-lesioned animals received total scores of 0, for no apparent cell loss in any of the kainic acid-lesioned animals’ neighboring brain regions, and thus no further analysis was conducted. For off-target transgene expression in the chronic chemogenetic inactivation experiments, every brain section was scored semiquantitatively, from 0 to 3 score (−, +, ++, +++) for each of the five neighboring brain regions. The scoring rubric was as follows: no mCherry+ cells (−); a minority of mCherry+ cells, ∼5–15 (+); a moderate number of mCherry+ cells, ∼15–100 (++); and a large number of mCherry+ cells, 100+ (+++). Each animal's off-target transgene expression score was averaged across each section containing a given off-target region, and these final values were analyzed.

#### Statistical analysis

**Figure 1.**
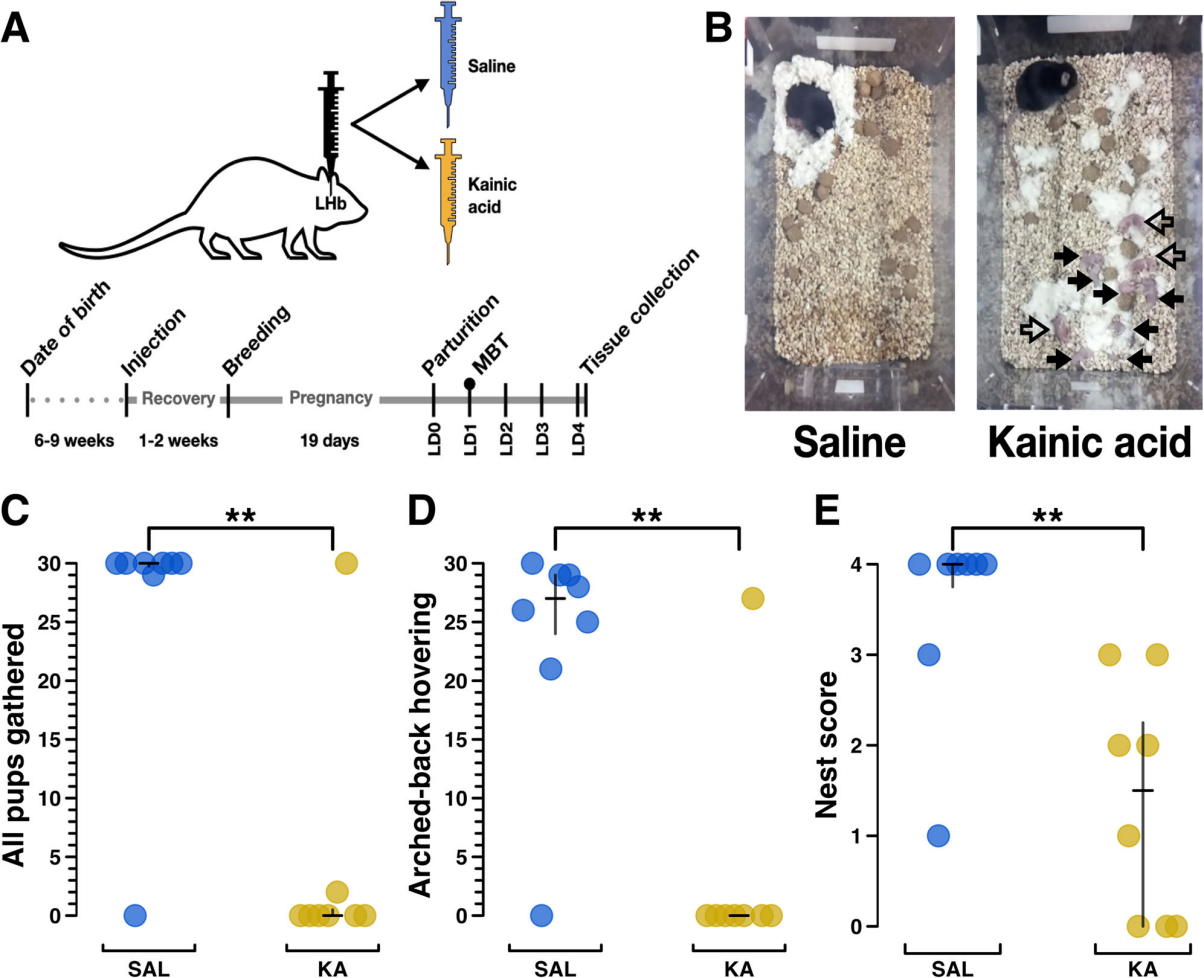
Kainic acid lesions of the LHb induce a severe maternal neglect phenotype in mice. ***A***, Experimental schematic of surgery/injection treatment (top) and timeline (bottom). Female mice were injected bilaterally with either saline or kainic acid into the LHb. These mice were bred following recovery, and after pregnancy and parturition, behavioral data were collected on LD1, denoted by a circle above LD1 on the timeline. The experimental endpoint occurred on LD4, when dams were killed so that their brains could be collected for histology. ***B***, Example still images showing the overhead view of a test cage containing a saline-treated and a kainic acid-treated female during a MBT. In the saline example, all pups are nested under the dam. In the kainic acid example, all pups are scattered. Filled arrows indicate dead pups and hollow arrows indicate living pups. ***C***, A plot from MBT showing the total number of scored video windows (out of 30) in which each dam, indicated as a separate plot point, was observed with all pups gathered, for saline treatment (*n* = 8) and kainic acid treatment (*n* = 8). Note that one kainic acid-treated mouse had no impairment in this score. Later histological analysis revealed that, for unknown reasons, this particular kainic acid injection failed to reduce the number or density of neurons in the LHb (Fig. 2). ***D***, A plot from MBT showing total number of scored video windows (out of 30) in which each dam was observed engaged in arched-back hovering over any pups (presumed nursing) or was visibly nursing. ***E***, A plot from MBT showing nest score (0, no nest attempted; 4, excellent nest; Numan and Callahan, 1980). Group differences were assessed using the Wilcoxon rank sum test except where otherwise stated, with significance levels of *p* < 0.05, *p* < 0.01, and *p* < 0.001 represented by *, **, and ***, respectively, here and for all subsequent figures. Median and IQR are represented by black bars, except where otherwise stated, here and for all subsequent figures. See Extended Data Figure 1-1 for further behavioral data and Movies 1 and 2 for example video clips from the saline group and kainic acid group MBTs, respectively.

**Figure 2.**
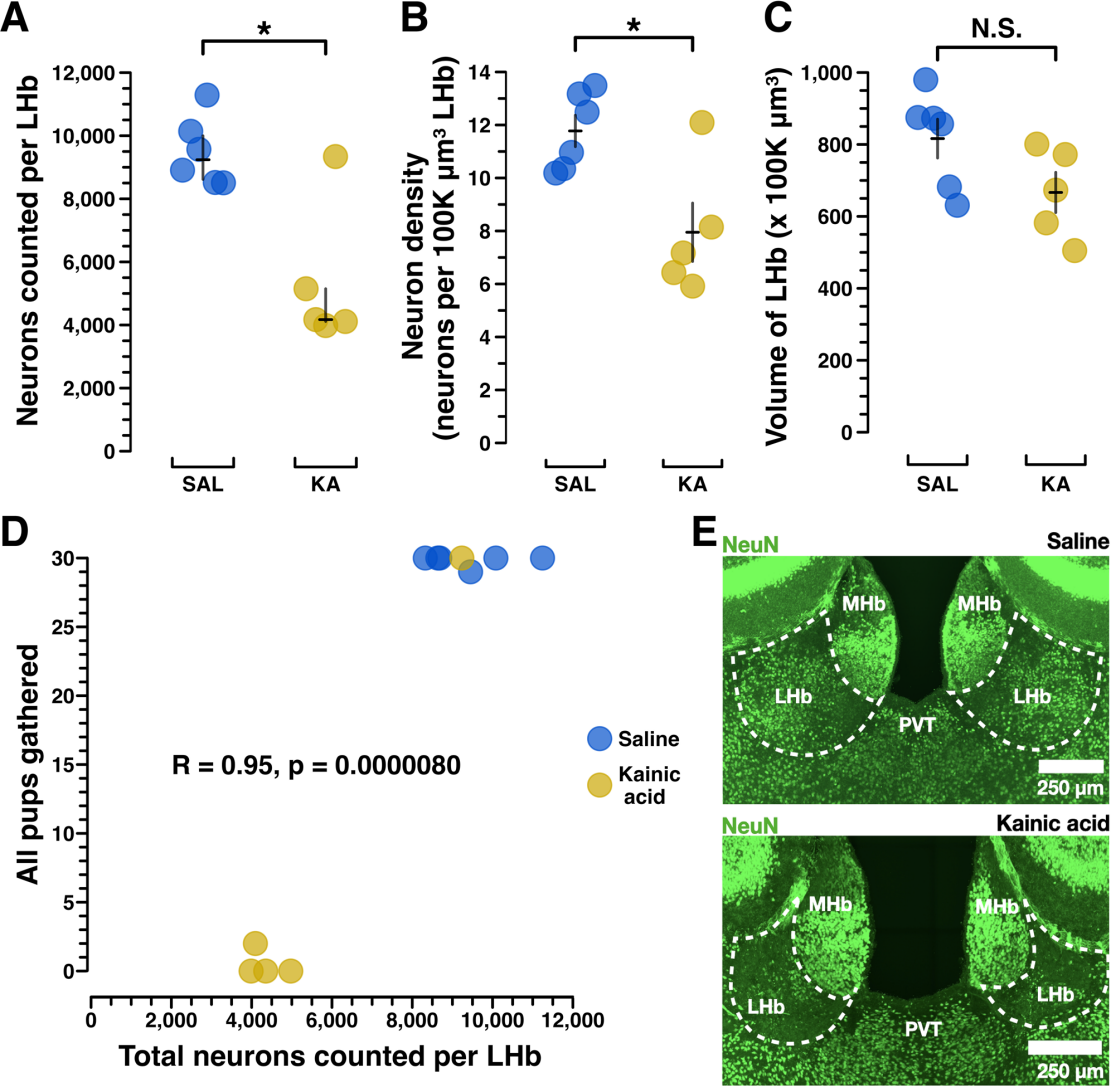
Kainic acid lesions reduced the number of LHb NeuN+ neurons. ***A***, Manual NeuN+ LHb neuronal counting was performed to validate the lesions caused by kainic acid treatment versus saline treatment. Manual counting of NeuN+ neurons across the entire extent of LHb shows a significant reduction of NeuN+ neurons in all but one kainic acid-treated mouse. In mouse X306 kainic acid treatment failed to reduce the number of NeuN+ LHb neurons. Importantly, mouse X306 also mothered her pups indistinguishably from the saline-treated group. Wilcoxon rank sum test of neurons counted per LHb, saline (*n* = 6) median (IQR) = 9,239 (8,615.75–9,998); kainic acid (*n* = 5) median (IQR) = 4,171 (4,111, 5,149); *p* = 0.0303. Black error bars depict the median and IQR. ***B***, To assess neuronal density, neurons per 100 K µm^3^ LHb, a standardized unit of volume, were calculated. These results show kainic acid lesion reduced the NeuN+ neuronal density in LHb. Welch's two-sample *t* test for normally distributed data, saline (*n* = 6) mean (standard error) = 11.78 (11.18, 12.37); kainic acid (*n* = 5) mean (standard error) = 7.95 (6.85, 9.05); *p* = 0.0212. Black error bars depict the mean and standard error. ***C***, Volumetric analysis of NeuN+ stained coronal sections across the A–P axis of LHb was conducted to see if total LHb volumes differed between groups, which could have occurred due to cell death following the lesion. Welch's two-sample *t* test for normally distributed data; saline (*n* = 6), mean (standard error) = 816.33 (762.33, 870.34); kainic acid (*n* = 5), mean (standard error) = 666.6 (610.70, 722.50); *p* = 0.0868. Black error bars depict the mean and standard error. ***D***, Pearson’s correlation between MBT score for the “all pups gathered” measure, and total neurons counted per LHb show that NeuN+ LHb neuron count is strongly correlated to behavioral performance of pup gathering. *R* = 0.95; *p* < 0.001. ***E***, Exemplar confocal images of coronal sections (approximately −1.50 mm posterior to the bregma) show NeuN+ LHb neurons in a saline-treated dam and a kainic acid-treated dam.

**Figure 3.**
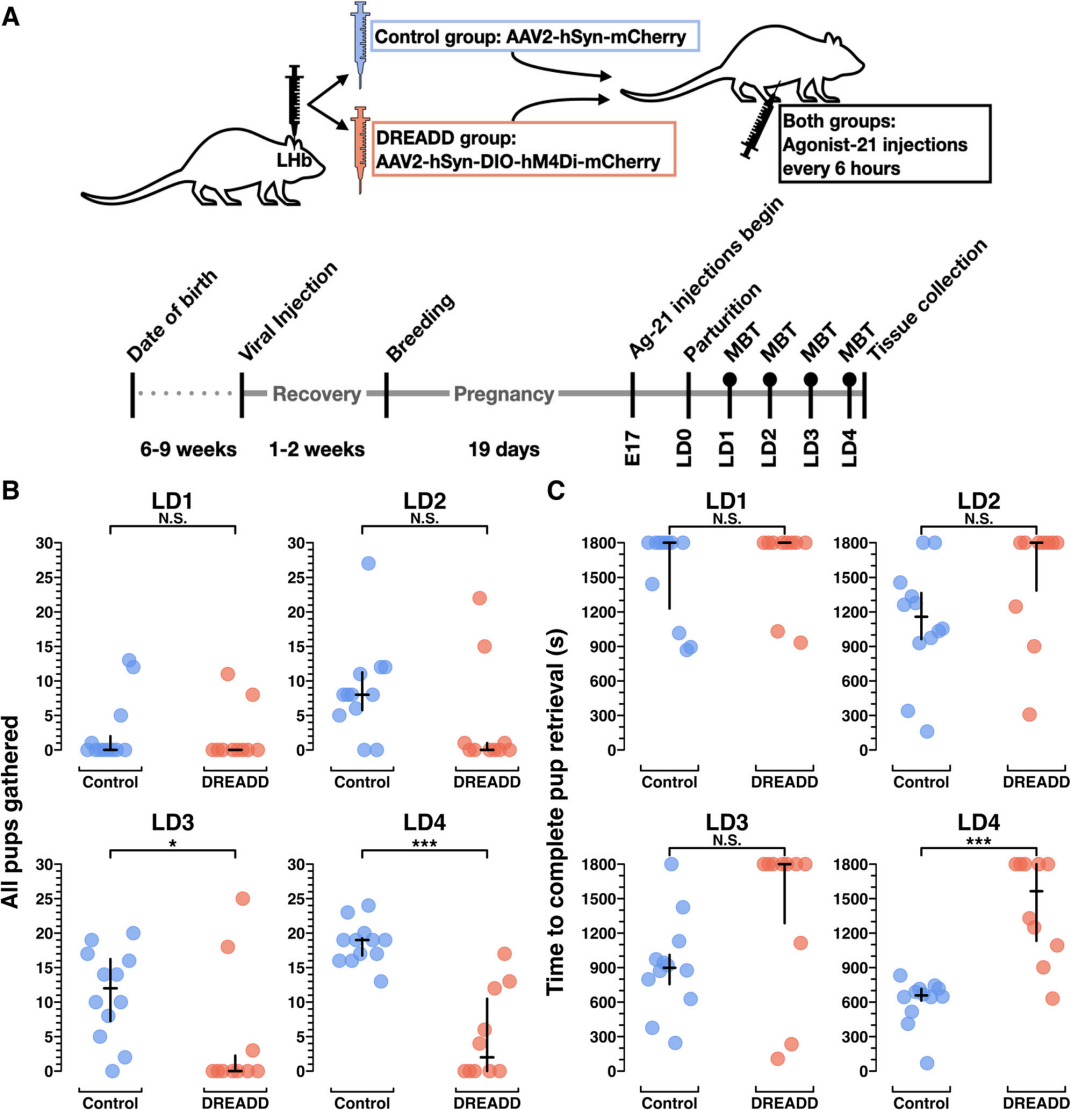
Chronic chemogenetic inactivation using DREADDs impairs the acquisition of pup retrieval, a novel maternal behavior, in primiparous dams. ***A***, Experimental schematic of treatment (top) and timeline (bottom). Filled circles above timeline points LD1–LD4 denote behavior data capture for all chronic chemogenetic inactivation data presented elsewhere. ***B***, A plot from MBTs LD1–LD4 showing total number of scored video windows (out of 30) in which each dam was observed with all pups gathered, after beginning the assay with all pups scattered. ***C***, A plot from MBTs showing the number of seconds elapsed (of an 1,800 s long assay) to the point when the dam had completed retrieval of all pups. Statistical comparisons were performed using the Wilcoxon rank sum test. See Extended Data Figure 3-1 for further behavioral data and Movies 3 and 4 for example video clips from the control group and the DREADD group, respectively.

**Figure 4.**
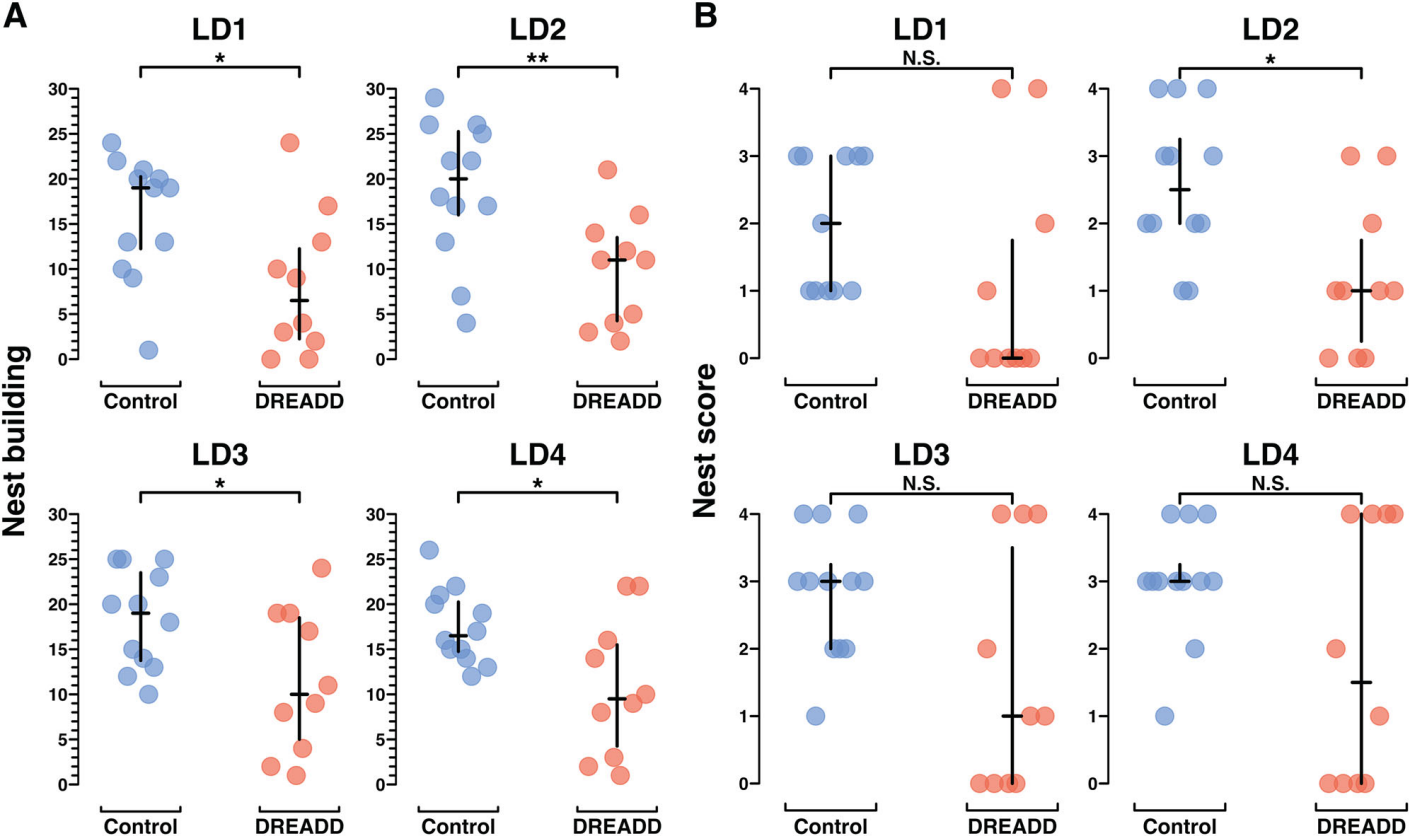
Chronic chemogenetic inactivation decreases nest building behavior in primiparous dams. ***A***, Plots from MBTs LD1–LD4 showing the total number of scored video windows (out of 30); each dam was observed engaged in nest building. Unlike pup retrieval, nest building is an established behavior in virgin female mice. ***B***, Plots from MBTs LD1–LD4 showing the nest score at 30 min. Statistical comparisons were performed using the Wilcoxon rank sum test. See Extended Data Figure 3-1 for further behavioral data.

**Figure 5.**
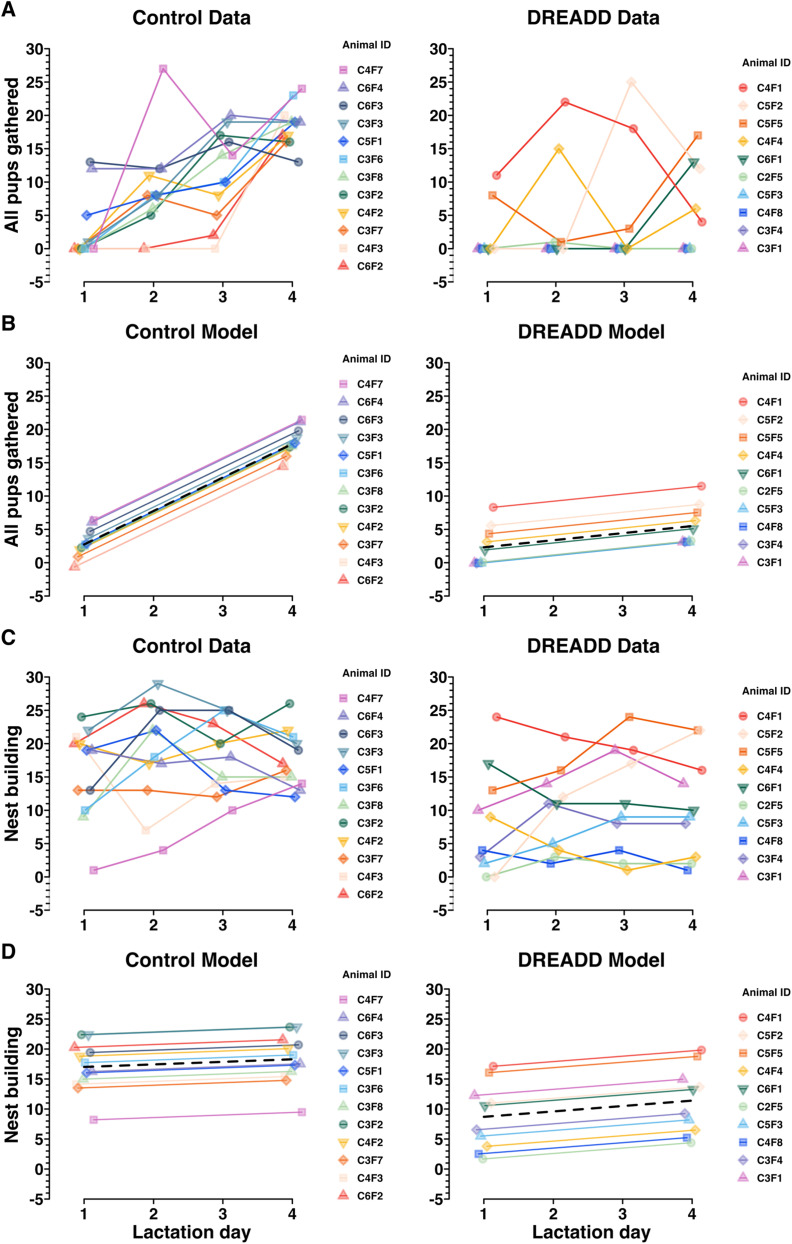
The effect of chronic chemogenetic inactivation on retrieval and nesting behaviors was compared across time and between groups, assessed using a random intercept linear mixed model. ***A***, Control data and DREADD data (also depicted in Fig. 3*B*) showing the effect of time on the “all pups gathered” variable in MBTs. A positive slope suggests that an animal acquired the novel maternal behavior of pup retrieval to gather all of her pups over the timescale of the experiments. ***B***, A random intercept linear mixed model depicting control group and DREADD group trajectories (black dashed lines) and individual dam trajectories across time for the “all pups gathered” variable. This model assesses whether the passage of time affects the groups differently on the “all pups gathered” variable (indicated by a difference between group slopes) and estimates the random individual differences for each animal. ***C***, Control data and DREADD data (also depicted in Fig. 4*A*) showing nest building MBT performance over the timescale of the experiments. ***D***, A random intercept linear mixed model depicting control group and DREADD group trajectories (black dashed lines) and individual dam trajectories for each group across time for the “nest building” variable. As expected, no effect of time on nest building behavior in MBTs was found. Since nest building is an established behavior in virgin female mice, the group intercept is of greater interest to assess a group difference in performance on the nest building variable. See Extended Data Figure 5-1 for exact term values, confidence intervals, degrees of freedom, *t* values, and significance testing.

**Figure 6.**
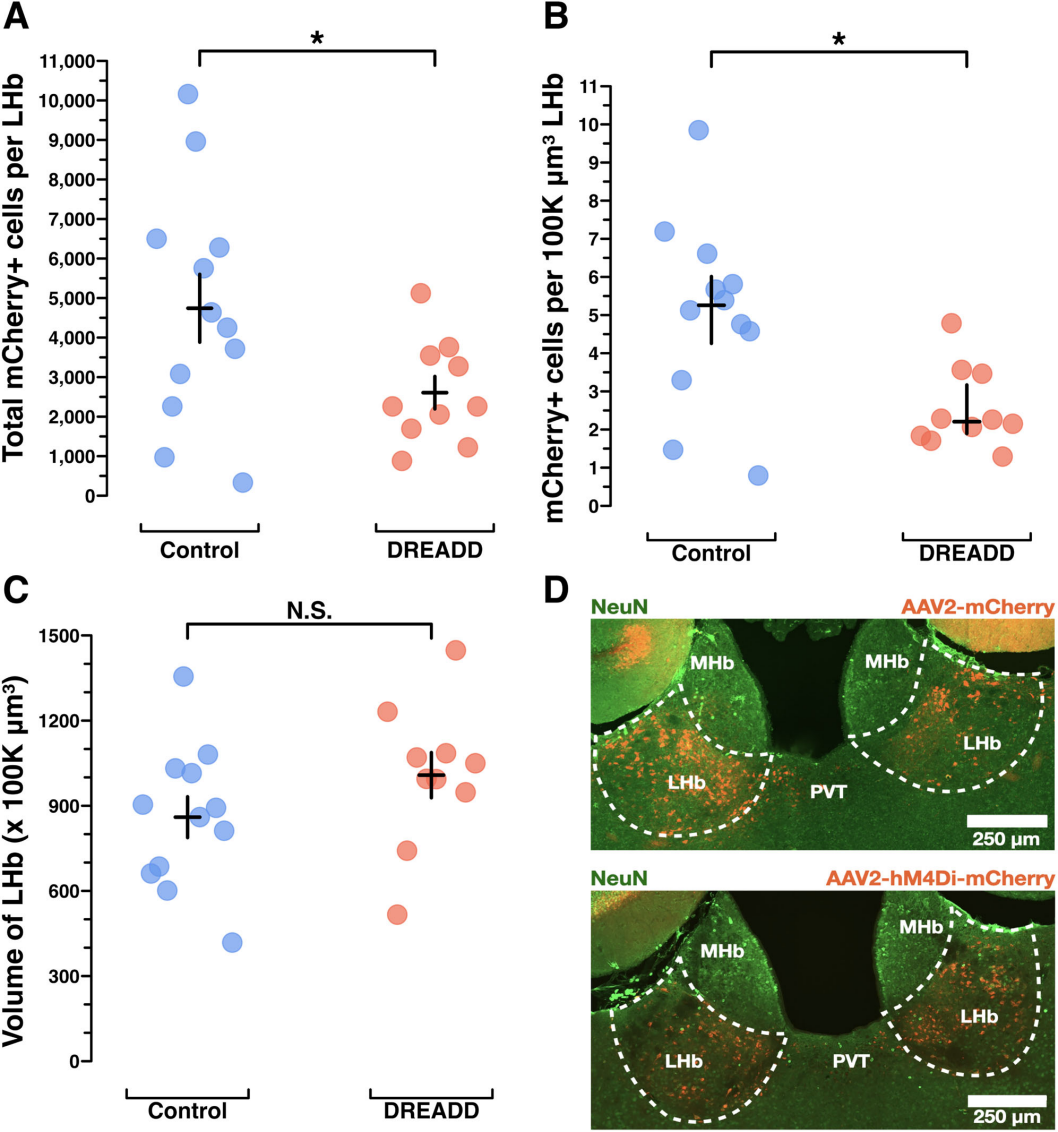
Histological analysis of mCherry immunoreactivity to assess DREADD transgene expression efficiency in dams’ brains following chronic chemogenetic inactivation behavior experiments. ***A***, Results from manual counting of mCherry+ cells across the entire extent of LHb show control dams likely had more mCherry+ LHb cells than DREADD-treated dams; see Results section for more details. Welch's two-sample *t* test, controls (*n* = 12) mean (standard error) = 4,741.83 (3,882.324, 5,601.342); DREADDs (*n* = 10) mean (standard error) = 2,606.8 (2,195.215, 3,018.385); *p* = 0.0400. ***B***, Results for mCherry+ cells per 100 K µm^3^ LHb as a standardized unit of volume show control dams had a higher density of mCherry+ LHb cells than DREADD-treated dams. Wilcoxon rank sum test with continuity correction, controls (*n* = 12) median (IQR) = 5.257 (4.256, 6.011); DREADDs (*n* = 10) median (IQR) = 2.205 (1.893, 3.169); *p* = 0.0169. ***C***, Results from manual volumetric analysis of coronal sections across the entire A–P axis of LHb to allow for density calculations in ***B***. Welch's two-sample *t* test, controls (*n* = 12) mean (standard error) = 859.88 (787.8498, 931.9168); DREADDs (*n* = 10); mean (standard error) = 1,007.826 (928.1617, 1,087.49); *p* = 0.1842. ***D***, Example histology of mCherry+ LHb cells in a control dam and a DREADD dam (approximately −1.50 mm posterior to the bregma). Statistical comparisons were performed using the Wilcoxon rank sum test. See Extended Data Figure 6-1 for Pearson’s correlations between “all pups gathered” and total mCherry+ cells per LHb in DREADD-treated dams and “nest building” and total mCherry+ cells per LHb in DREADD-treated dams, which found no correlation. See Extended Data Figure 6-2 for Spearman correlations of the magnitude of mCherry+ off-target transgene expression in neighboring brain regions in DREADD-treated dams and their “all pups gathered” and “nest building” performance; no correlations were found.

**Figure 7.**
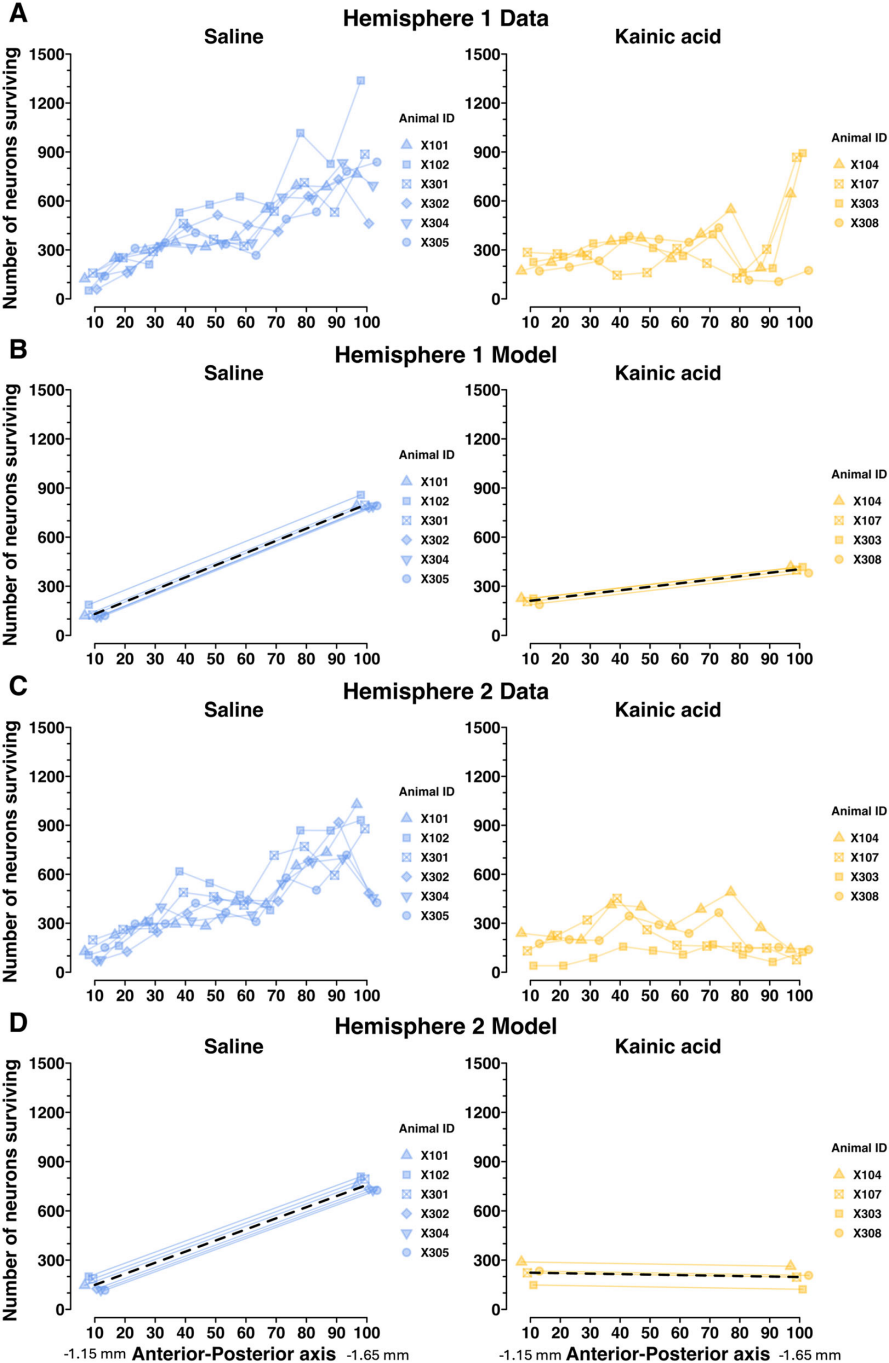
The LHb neurons regulating maternal behavior may preferentially reside in the posterior LHb, assessed using a random intercept linear mixed model. Here, we test whether the spatial distribution of remaining neurons along the A–P axis of LHb is significantly related to maternal behavior outcomes. ***A***, NeuN+ LHb neuron counts in Hemisphere 1 for each decile of the AP axis of LHb. ***B***, A random intercept linear mixed model depicting saline-treated and kainic acid-treated group trajectories, and individual dam trajectories, for NeuN+ LHb neuron counts in Hemisphere 1 across the A–P axis. This model, along with that in ***D***, shows a statistically significant relationship between maternal behavior outcomes and LHb neuron counts by A–P axis decile and suggests that the maternally relevant LHb neurons may reside at the posterior aspect of LHb. ***C***, NeuN+ LHb neuron counts in Hemisphere 2 for each decile of the AP axis of LHb. ***D***, A random intercept linear mixed model depicting saline-treated and kainic acid-treated group trajectories, and individual dam trajectories, for NeuN+ LHb neuron counts in Hemisphere 2 across the A–P axis. This again shows a statistically significant relationship between maternal behavior outcomes and LHb neuron counts by A–P axis decile, particularly suggesting the posterior aspect of LHb may be the most important for maternal behavior. Bregma values for the 10th and 100th percentiles of the A–P axis printed under the *x*-axis. See Extended Data Figure 7-1 for model terms, confidence intervals, degrees of freedom, *t* values, and significance testing. See Extended Data Figure 7-2 for depiction of the data for Animal X306 who was excluded from Figure 7 and Extended Data Figure 7-1.

R Studio (version 2023.09.0 + 463) was used with R Project for Statistical Computing (RRID: SCR_001905, version 4.3.1) for analysis. All error bars in the figures show the median and interquartile range (IQR) or the mean and standard error, as stated in the figure legend. All univariate behavior analyses are nonparametric Wilcoxon rank sum tests. Histology tests in Figures 2 and 6 are either Wilcoxon rank sum tests or Welch's two-sample *t* tests, depending on data normality. Pearson’s correlations were used to compare total neuron counts/total transgene-expressing cell counts to behavior measures (Fig. 2*D*; Extended Data Fig. 6-1). Spearman correlations were used to test for correlation between behavior results and nonlinear scale histology data from off-target brain region transgene expression (Extended Data Fig. 6-2). Data normality was assessed using the Shapiro–Wilk test, *Q*-*Q* plots, and density histograms, data that were borderline normal/non-normal distributions were tested nonparametrically. The random intercept linear mixed models were all conducted using the R package: lme4 (RRID: SCR_015654). All statistical tests, particularly the linear mixed models, were conducted in direct consultation with the Johns Hopkins Biostatistics Center. The random intercept models were tested for Akaike Information Criterion (AIC) value against multivariate fixed-effect linear models and univariate linear models. In all models presented, the random intercept model was the most parsimonious, with the lowest AIC score. All analysis was conducted on a Mac-Mini (3.2 GHz 6-Core Intel Core i7, running macOS Ventura 13.2). See Table 1 for a list of all statistical testing conducted in Figures 1–7.

#### Data availability

All the data presented in this manuscript, along with all of the code for the multilevel multivariate models in Figures 5 and 7 are made available at https://github.com/ghost-pants/multilevel-modeling-code. To run the code, please read the included readme.txt file.

## Results

### LHb kainic acid lesions induced a severe maternal neglect phenotype in dams

Seeking to extend prior findings that kainic acid lesions of the LHb prevented the onset of maternal behavior in the rat (Matthews-Felton et al., 1995), we tested the effects of kainic acid LHb lesions in the mouse dam. Pup-naive virgin female mice received bilateral LHb injections of either saline or kainic acid. Following recovery, breeding, and pregnancy, the mice were moved to individual behavior boxes with continuous video monitoring on E17 to habituate prior to parturition (Benedict and Cudmore, 2023; Fig. 1*A*). While standard MBTs were planned to begin on LD2, half of all dams’ (8/16) litters had died by then. Instead, naturalistic MBTs (Carcea et al., 2021; Dvorkin and Shea, 2022; Schuster et al., 2023) were scored from footage obtained on LD1 in which all dams showed activity. Naturalistic MBT analysis showed that 7/8 kainic acid-treated dams failed to engage in any maternal behavior, resulting in the death of 100% of their litters by LD2. Importantly, dams did not cannibalize their pups but left them strewn about the cage as if they were part of the environment, occasionally sniffing or stepping on them (Fig. 1*B*; Movie 2). The neglected pups exhibited no visible milk spots (Fig. 1*B*; Extended Data Fig. 1-1). Naturalistic MBTs do not begin by manually scattering the pups to specifically test pup retrieval, but pups do intermittently fall out of the nest. The best proxy variable for retrieval behavior is “all pups gathered” (Fig. 1*C*), which shows that seven of eight saline-treated dams had all of their pups gathered in nearly all of the video windows that were scored. Simultaneously, seven of eight of the kainic acid-treated dams failed to have all their pups gathered in nearly all the scored video windows (*p* < 0.01; Wilcoxon rank sum test in all comparisons, unless otherwise stated). Arched-back hovering/visible nursing scores (*p* < 0.01; Fig. 1*D*) were similar to “all pups gathered.” Nest scores (Fig. 1*E*) show that most saline-treated dams built excellent nests with high structured walls and used all their available nesting material, whereas kainic acid-treated dams’ nests scored far worse (*p* < 0.01; see Materials and Methods, Behavior scoring for more detail on nest scoring). For a table of all behaviors measured, see Extended Data Figure 1-1. For example video footage of saline-treated and kainic acid-treated dams, see Movies 1 and 2.

10.1523/ENEURO.0092-24.2024.f1-1 Figure 1-1 **Table showing all the behavioral results from the kainic acid lesion experiments.** Medians and IQRs are provided by group, and p-values were calculated using the Wilcoxon rank sum test. P-values were left unadjusted for multiple comparisons in concordance with the recommendation from the Johns Hopkins Biostatistics Center. Note the difference in scale from 0-30 (variables 1-9), and variable 10 (nest score), which is on a 0-4 scale. See the Methods section for further details on the nest score scale. ‡ Pup interactions include pup sniffing, inspecting, anogenital licking, or retrieval. Download Figure 1-1, TIF file.

### Lesion validation

Immunohistochemistry for NeuN enabled exhaustive manual counting of LHb neurons. This showed that saline-treated dams had about twice the LHb neurons of those dams treated with kainic acid [saline (*n* = 6) median (IQR) = 9,239 (8,615.75–9,998), kainic acid (*n* = 5) median (IQR) = 4,171 (4,111–5,149); *p* < 0.05; Fig. 2*A*,*E*, example images]. Neuronal density showed a similar pattern, with saline-treated dams having two times the neuronal density of kainic acid-treated dams [saline (*n* = 6) mean (standard error) = 11.78 (11.18–12.37), kainic acid (*n* = 5) mean (standard error) = 7.95 (6.85–9.05); *p* < 0.05; Fig. 2*B*]. While a reduction in LHb volume would not be surprising given the neuronal death associated with kainic acid lesion, any such difference did not reach statistical significance [saline (*n* = 6), mean (standard error) = 816.33 (762.33–870.34), kainic acid (*n* = 5), mean (standard error) = 666.6 (610.70–722.50); *p* = 0.086; Fig. 2*C*].

Notably, the only kainic acid-treated dam that performed maternal behavior at control levels (mouse X306) was later shown to have the same number of total remaining neurons as those from the saline-treated group (Fig. 2*A*,*B*; Extended Data Fig. 7-2). The only saline-treated dam that neglected her pups (Fig. 1*C*–*E*) may have reflected the known baseline rate of neglect in mouse dams (Schuster et al., 2023), as her brain could not be assessed for possible accidental injection damage, since the brain tissue from five animals (two saline-treated and three kainic acid-treated), including hers, was damaged during COVID-19 lab shut downs, and the subsequent histology was uninterpretable.

Using behavioral results from only the brains with complete neuronal count data, we performed a Pearson’s correlation of dams’ “all pups gathered” scores, and their total neurons were counted per LHb (Fig. 2*D*). This showed a correlation coefficient of *r* = 0.95 (*p* < 0.0001), suggesting a strong positive correlation between a dam's remaining LHb neuron count and her maternal behavior performance.

### Chronic chemogenetic LHb inactivation using DREADDs

The results from the kainic acid lesion experiments left open the possibility that LHb lesions blocked normal neuroplastic changes during pregnancy that are required for maternal behavior onset but that the LHb may not be necessary for ongoing maternal behavior in the primiparous mouse dam. We sought to temporally refine the LHb manipulation through the use of inhibitory DREADDs. DREADDs are often used for cross-over experimental designs, wherein all animals are injected with a DREADD-expressing virus, but injection treatment is alternated each day within animals between saline and a DREADD-agonist. However, maternal behavior is ill-suited to alternating saline days with DREADD-agonist days, since there are multiple latent variables, namely, hormonally mediated maternal behavior onset, learned maternal behavior acquisition, and changing pup needs, operating on a multiday timescale in the early postpartum period. Thus, we opted for chronic LHb inactivation (Benoit et al., 2022; Canetta et al., 2022; Tiwari et al., 2022) and injected the LHb of half the animals with an active inhibitory-DREADD virus [AAV2-hSyn-hM4D(Gi)-mCherry] and the other half with a control virus (AAV2-hSyn-mCherry). From E17 to LD4, all mice received four times daily injections with Agonist-21, a designer ligand for the hM4D(Gi) metabotropic receptor (Fig. 3*A*). Agonist-21 does not back-convert into psychoactive clozapine and has been shown to induce statistically significant behavioral effects as a result of DREADD agonism for 6 h (Thompson et al., 2018; Jendryka et al., 2019; Ferrari et al., 2022). By administering Agonist-21 to both groups, the control group helps to control for the possibility of unknown off-target effects of Agonist-21.

10.1523/ENEURO.0092-24.2024.f3-1 Figure 3-1 **Table showing all the behavioral variables from chronic chemogenetic inactivation experiments.** Medians and IQRs are provided by group for LD1-LD4, and p-values were obtained by the Wilcoxon rank sum test. P-values are left unadjusted for multiple comparisons, in concordance with the recommendation from the Johns Hopkins Biostatistics Center, since all comparisons were planned prior to data collection, comparisons were made to test specific hypotheses, and the number of comparisons were on the order of dozens, not thousands. Note the difference in scale from 0-30 (variables 1-9), and variables 10-12 (0-1800, 0-1800, and 0-4 scales respectively). See Methods section for further detail on the scales. Download Figure 3-1, TIF file.

### Chronic LHb inactivation impairs the acquisition of pup retrieval, a novel maternal behavior in primiparous dams

Since all dams in these experiments were pup-naive until parturition, retrieving manually scattered pups to the nest was a novel behavior. Both control group dams and DREADD group dams were slow to retrieve their manually scattered pups on LD1 (*p* = 0.53), but by LD2, control group dams were already improving, while DREADD group dams performed similarly to the day before. By LD4, control group dams had learned to retrieve all of their pups at least two to three times faster than DREADD groups dams (*p* < 0.001; Fig. 3*B*,*C*; Extended Data Fig. 3-1). On LD4, DREADD dams were divided bimodally, with five DREADD dams still failing to complete pup retrieval at all during the 30 min assay (and thus receiving a score of 1,800) and the other five DREADD dams eventually completing pup retrieval, yet doing so much slower than the control group dams (*p* < 0.001; Fig. 3*C*; Extended Data Fig. 3-1). For example videos of LD4 behavior assays, see Movies 3 and 4. Altogether, these data suggest chronic LHb inactivation blocked acquisition of the novel pup retrieval behavior completely in about half the DREADD group and at minimum impaired acquisition in all but two of the DREADD females (*n* = 10) by LD4 (Figs. 3*B*,*C*, 5*A*).

### Chronic LHb inactivation reduces nest building, an established behavior in primiparous dams

Unlike rats, virgin adult mice spontaneously build nests (Oortmerssen, 1971; Van Loo and Baumans, 2004). As such, primiparous mouse dams do not need to learn to build nests. However, since highly motivated nest building and high nest scores in pregnant female mice predict engagement in other maternal behaviors as well as pup survival (Schuster et al., 2023), nest building motivation is an important maternal behavior variable.

Across all days, control group dams spent significantly more time engaged in nest building than DREADD group dams (Fig. 4*A*; Extended Data Fig. 3-1). Control group dams also built nests more reliably than DREADD group dams, with higher overall nest scores (Fig. 4*B*; Extended Data Fig. 3-1). However, similar to pup retrieval, DREADD dams’ nest scores on LD4 were bimodal, with about half the DREADD group females receiving high nest scores and the other half failing to build a nest at all. These results suggest that for about half the DREADD group, LHb inactivation severely impaired nest building motivation.

### Linear mixed model of a novel versus an established maternal behavior in primiparous mouse dams

Since each univariate analysis presented in Figures 3 and 4 is analyzed in isolation of any other lactation day, significance testing only assesses group differences at that individual time point. Since what we could gather visually from Figure 3, that there appeared to be impaired learning of pup retrieval in DREADD group females, was not being tested by the Wilcoxon rank sum tests, we sought a superior statistical approach. Specifically, we sought an approach that would enable analysis of each dam's individual longitudinal performance, nested within her treatment group identity. In consultation with the Johns Hopkins Biostatistics Center, we conducted a random intercept analysis, a type of random effect linear mixed model (Verbeke, 1997; Barr, 2013; Bates et al., 2015). Random intercept models enable analysis of multivariate interactions between the treatment group and behavior variables, across time or space, which enables each datapoint to be linked to the individual animal to which it belongs. The model has the fixed effects, which are the effects of the parameters of the experiment (i.e., “all pups gathered,” lactation day, treatment group, and animal ID for Fig. 5*B*). As an additional improvement over univariate methods, the model also has random effects (i.e., individual animal IDs) introduced by random individual differences between mice or their particular injection surgeries. This enables contextualization of the effect size of the tested interaction, in comparison with any random effects introduced by nonindependent terms. This form of analysis is widely used in adjacent fields and, based on conversations with several biostatisticians, ought to be being used every time, for example, results from multiple cohorts of animals are analyzed together. Put another way, anytime there is hierarchical structure to data, like if thousands of cells are sequenced across 12 animals, those data need to be pooled in a manner accounting for the nonindependence of the data from each animal.

10.1523/ENEURO.0092-24.2024.f5-1 Figure 5-1 **Table of values plotted in Figure 5.** Table showing the Control and DREADD-treated group intercepts and group slopes for the random intercepts model depicted in Figure 5. For the “all pups gathered” variable, a near-zero intercept for both control and DREADD groups suggests a novel behavior on LD1, and a steep positive slope suggests rapid acquisition of the pup retrieval behavior for the control group. The much smaller slope for the DREADD group suggests their treatment impaired pup retrieval learning. For the “nest building” variable, the near-zero slopes for both groups are consistent with an established behavior. The much lower group intercept for the DREADD group suggests nest building performance was impaired by their treatment. Confidence intervals, degrees of freedom, *t* values, and significance testing for each term is shown in subsequent columns. Significance testing for the control group rows in this table reflect the model’s confidence that the true term is nonzero, and the significance testing for the DREADD group rows in this table reflect the model’s confidence that there are statistically significant differences between the control group and the DREADD group. Download Figure 5-1, TIF file.

In Figure 5*A*, the raw behavior data for “all pups gathered” shows each dams’ performance for each MBT (LD1–LD4), with one plot for each treatment group. Those same data are then modeled in Figure 5*B*, where the random intercept model finds a statistically significant (*p* < 0.001) difference between the slopes for each group (Extended Data Fig. 5-1). No significant difference is found between group intercepts (*p* = 0.26), suggesting there is no initial difference in retrieval motivation or performance between control and DREADD groups but only the difference that emerges over time, as the control group dams learn to conduct pup retrieval while the DREADD group dams’ pup retrieval learning is impaired (Fig. 5; Extended Data Fig. 5-1).

In Figure 5, *C* and *D*, the raw data and modeled data are shown for nest building performance over time. In contrast to the random intercept model of retrieval, nesting behavior is already established and thus both groups have very subtle slopes with no between group difference (*p* = 0.57). There is, however, a statistically significant difference in group intercepts, with the control group engaging in twice as much nest building as the DREADD group, across time (*p* < 0.01; Fig. 5*D*; Extended Data Fig. 5-1). This difference suggests an intergroup difference in baseline nest building motivation.

### mCherry immunoreactivity in dams’ brains following chronic chemogenetic inactivation behavior experiments

We sought to confirm viral injection targeting and to assess whether maternal behavior was differentially impacted by the number of mCherry+ LHb cells expressing DREADDs. mCherry+ LHb cells were exhaustively counted across both groups, and total LHb volumes were measured. The total number of mCherry+ cells in LHb was higher for the control group than the DREADD group [controls (*n* = 12) mean (standard error) = 4,741.83 (3,882.32–5,601.34), DREADDs (*n* = 10) mean (standard error) = 2,606.8 (2,195.22–3,018.39); *p* < 0.05; Fig. 6*A*]. The control group had higher mCherry+ cell density than the DREADD group, calculated as mCherry+ cells per 100 K µm^3^ LHb [controls (*n* = 12) median (IQR) = 5.257 (4.256–6.011), DREADDs (*n* = 10) median (IQR) = 2.205 (1.893–3.169); *p* < 0.05; Fig. 6*B*]. LHb total volumes in 100 K µm^3^ were not statistically different between groups [controls (*n* = 12) mean (standard error) = 859.88 (787.85–931.92), DREADDs (*n* = 10), mean (standard error) = 1,007.83 (928.16–1,087.49); *p* = 0.18; Fig. 6*C*]. This difference in transgene expression is unsurprising given the difference in the insert size between the two viruses (controls virus, 711 basepairs; DREADD virus, 2,169 basepairs) and the susceptibility of adeno-associated viruses (AAVs) to reduced transgene expression efficiency with larger inserts (Hermonat et al., 1997). Counter to the results seen in the kainic acid lesion validation data (Fig. 2), the number of mCherry+ LHb cells did not predict LD4 performance in DREADD group dams for either pup retrieval (*R* = 0.0019; *p* = 0.9580; Extended Data Fig. 6-1*A*) or nest building (*R* = 0.086; *p* = 0.8140; Extended Data Fig. 6-1*B*). In fact, many of the dams with severely impaired pup retrieval had quite modest LHb transgene expression (Extended Data Fig. 6-1*A*,*B*). This finding raises the questions of whether a neighboring brain region with off-target DREADD expression could be predictive of maternal behavior, whether AAV-hSyn-hM4Di-mCherry was inhibiting neural activity in LHb in the DREADD group dams, and whether particular LHb subregions or cell types may be most relevant for maternal behavior regulation.

10.1523/ENEURO.0092-24.2024.f6-1 Figure 6-1 **mCherry** **+** **LHb cell counts do not predict maternal behavior outcomes in DREADD-treated dams.** A) Pearson correlation between MBT score for the “all pups gathered” variable (out of 30) and total + mCherry cells counted per LHb in DREADD-treated dams shows no correlation (R = 0.0019, *p* = .9580). B) Pearson correlation between MBT score for “nest building” (out of 30) and total mCherry + cells counted per LHb in DREADD-treated dams shows no correlation (R = 0.086, *p* = .8140). The number of mCherry + cells in the LHb does not appear to predict maternal behavior outcomes in DREADD-treated dams. Download Figure 6-1, TIF file.

10.1523/ENEURO.0092-24.2024.f6-2 Figure 6-2 **Off-target transgene expression in neighboring brain regions do not predict maternal behavior in DREADD group dams.** The five brain regions most closely bordering LHb were subjectively blind-scored for transgene expression magnitude (-, +, ++, +++) (see methods section for rubric) on each brain section for each DREADD-treated dam. The scores, produced by a single scorer, were totaled across all sections for each dam, then a Spearman correlation was performed comparing “all pups gathered” and “nest building” scores against total transgene expression scores for each off-target region. No regions were statistically significant. Download Figure 6-2, TIF file.

To address the question of whether off-target transgene expression predicts maternal behavior in DREADD group dams, a blinded scorer assigned subjective scores to each brain section of the hippocampus, medial habenula, paraventricular nucleus of the thalamus, central lateral nucleus of the thalamus, and anterior pretectal nucleus, as these were the only brain regions with off-target mCherry+ cells. Spearman correlations showed none of the off-target regions’ transgene expression magnitude was related to DREADD dams’ performance on LD4 for “all pups gathered” or “nest building” (Extended Data Fig. 6-2).

To confirm that chronic Agonist-21 injection and AAV-hSyn-hM4Di-mCherry infection suppressed neural activity in LHb, we conducted blinded exhaustive cell counting of c-Fos+ cells in the LHbs of control group and DREADD group dams (Extended Data Fig. 6-3). We found a statistically significant reduction in LHb c-Fos expression in the DREADD group (*p* = 0.011; Wilcoxon rank sum test; Extended Data Fig. 6-3*A*). Simultaneously, there was no significant difference in LHb volumes between groups (*p* = 0.69; Wilcoxon rank sum test; Extended Data Fig. 6-3*B*), which further validates the cell density calculations. Additionally, we noticed that the DREADD animals that failed to perform any pup retrieval on LD4 (immediately prior to being killed/brain tissue collected) also had the lowest c-Fos+ cell density in LHb of any of the animals. Similarly, two of the three best DREADD retrievers also had the two highest c-Fos+ cell densities of the DREADD group. These observations are quantified with a Spearman correlation between the retrieval scores for LD4 and the c-Fos+ cell density per LHb across both groups (*R* = 0.61; *p* < 0.01; Extended Data Fig. 6-3*C*). Altogether, these data support the conclusion that chronic administration of Agonist-21 lowered the neuronal activity in DREADD group LHbs and that the decreased neuronal activity in the LHb likely underlies the maternal behavior deficit displayed by the DREADD group.

10.1523/ENEURO.0092-24.2024.f6-3 Figure 6-3 **LHb c-Fos expression following LD4 experiments reduced in DREADD group as compared to control group.** Manual counting of c-Fos + cells per 100  K µm^3^ LHb show control dams had higher cell density of c-Fos + cells in LHb following LD4 behaviors testing than DREADD dams. Wilcoxon rank sum test, controls (n = 10) median (IQR) = 1.893 (1.280-2.221), DREADDS (n = 8) median (IQR) = 1.013 (0.819-1.202), *p* = .0117. B) Measuring normalized LHb volume for control dams versus DREADD dams shows no significant difference in volumes. Total LHb volumes per animal were divided by the number of LHb sections available for quantification per animal to obtain the normalized LHb volume. Wilcoxon rank sum test, controls (n = 8), median (IQR) = 62.85 (60.80-74.69), DREADDS (n = 8) median (IQR) = 72.75 (54.72-76.50), *p* = .6965. C) Results from a Spearman correlation test show a significant positive correlation between the retrieval scores for LD4 and the c-Fos + cell density per LHb across both groups (R = 0.61, *p* < .01). Download Figure 6-3, TIF file.

### Spatial histology random intercept model

To partially answer the question of whether particular LHb subregions may be most relevant for maternal behavior, we performed another random intercept model, this time of the kainic acid lesion histology data (Fig. 7). Since we sought to test whether the spatial distribution of remaining neurons along the anterior–posterior (A–P) axis differed by maternal behavior outcome, we excluded dam X306 (the single kainic acid-treated dam who mothered her pups, Figs. 1*C*–*E*, 2*D*) from this analysis so that within-group maternal behavior outcomes were homogenous. This was done in concordance with the recommendations from the Johns Hopkins Biostatistics Center.

10.1523/ENEURO.0092-24.2024.f7-1 Figure 7-1 **Table of specific term values plotted in Figure 7.** The group terms “10 percentile” and “100^th^ percentile” refer to the value of the y-value at those deciles, in this case the number of neurons remaining in the first and tenth deciles of the AP axis. Significance testing in all rows labeled “saline” represent the model’s certainty that the saline group’s true term value (10^th^ or 100^th^ percentile y-value, or slope) is nonzero, while the “kainic acid” rows’ statistical significance column denote the model’s certainty that there is a statistically significant difference between the saline and kainic acid groups. The model fails to find a significant difference between saline and kainic acid group y-values at the 10^th^ percentile, while finding a statistically significant difference at the 100^th^ percentile. This means the posterior aspect of LHb may be where the maternally relevant neurons reside. Slopes provided in the table are computed as if the first decile is the intercept, and thus a large positive group slope, like that of the saline group, means there were many neurons at the posterior aspect of LHb. The comparatively slight (Hemisphere 1) or negative (Hemisphere 2) group slope for the kainic acid group represents their relative paucity of neurons remaining in posterior LHb. Download Figure 7-1, TIF file.

10.1523/ENEURO.0092-24.2024.f7-2 Figure 7-2 **Spatial histology comparison of Animal X306’s remaining neuron counts across AP axis of LHb.** X306 received kainic acid treatment but mothered her pups indistinguishably from the saline group, and her posterior LHb appears intact. A) NeuN + LHb neuron counts in Hemisphere 1 for each decile of the AP axis of LHb for the saline group, the kainic acid group, and animal X306 (left, middle, right, respectively). B) NeuN + LHb neuron counts in Hemisphere 2 for each decile of the AP axis of LHb for the saline group, the kainic acid group, and animal X306 (left, middle, right, respectively). Download Figure 7-2, TIF file.

**Movie 1.** Example video clip of a saline-treated dam. An example video clip from a naturalistic MBT conducted on LD1. [View online]

**Movie 2.** Example video clip of a kainic acid-treated dam. An example video clip from a naturalistic MBT conducted on LD1. [View online]

**Movie 3.** Example video clip of a control group dam. The first 2.5 min of the LD4 MBT in a control group dam. [View online]

**Movie 4.** Example video clip of a DREADD group dam. The first 2.5 min of the LD4 MBT in a DREADD group dam. [View online]

With the remaining 10 dams’ histology data (*n* = 4 kainic acid; *n* = 6 saline), we segmented remaining neuron counts by decile along the A–P axis. The 10th percentile marks the most anterior tenth of LHb sections quantified (approximately −1.15 mm from the bregma), and the 100th percentile marks the most posterior tenth of LHb sections quantified (approximately ∼1.65 mm from the bregma; Fig. 7; Extended Data Fig. 7-1). Each hemisphere was analyzed independently. The spatial histology data for Hemisphere 1 and Hemisphere 2 are presented in Figure 7, *A* and *C*, respectively. Those same data are then modeled in Figure 7, *B* and *D*. The random intercept model finds no statistically significant difference between groups at the 10th percentile AP mark (*p* = 0.17 and *p* = 0.19 for Hemispheres 1 and 2) but finds a significant difference between groups at the 100th percentile A–P mark and between group slopes from 10th to 100th percentiles, for both hemispheres (*p* < 0.001 for 100th percentile mark and for group slopes, for both hemispheres; Extended Data Fig. 7-1). In the table presented in Extended Data Figure 7-1, significance testing for saline-treated group–term values (i.e., intercepts at 10th and 100th percentile A–P mark and group slope) reflects the model's confidence that the true saline group term value is nonzero, whereas the significance testing for the kainic acid-treated group terms reflect the model's confidence that there is a significant difference between saline and kainic acid groups. This is all to say, the results of the significance testing in Extended Data Figure 7-1 that tests for differences between the two groups are the *p* values printed in the rows for labeled “Kainic acid” group. These findings suggest that remaining neuron counts in the posterior LHb, and not the anterior LHb, are predictive of maternal behavior outcomes.

Animal X306 was kainic acid-treated but went on to mother her pups indistinguishably from dams in the saline-treated group. Her total remaining LHb neuron count was also indistinguishable from those of the saline group (Fig. 2*A*). However, upon reviewing her spatial histology, X306 has lower neuron counts in the anterior-most deciles of LHb compared with saline dams. This could mean her kainic acid injection failed, and her anterior LHb neuron counts are the result of normal variation, or her lesion had an anterior LHb bias, thus sparing the central and posterior LHb. She displayed intact maternal behavior. Although no conclusions can be drawn from her single case, X306's spatial histology and maternal behavior outcome support our principal spatial histology finding that an intact posterior LHb enables maternal behavior. We present her individual spatial histology data separately for comparison (Extended Data Fig. 7-2).

## Discussion

The goal of the present study has been to determine whether the LHb is required for maternal behavior in the naturally parturient primiparous mouse dam. We found that lesioning the LHb with kainic acid induced a severe maternal neglect phenotype in the mouse dam. Lesioned dams ignored their pups completely, failing to ever feed or retrieve them to the nest. Because of this neglect, the pups all died within 60 h of birth (Fig. 1; Extended Data Fig. 1-1, Movies 1 and 2).

To test the hypothesis that ongoing LHb activity is required for maternal behavior, we chronically inactivated LHb using DREADDs starting just before parturition. This experiment helped exclude the possibility that kainic acid lesions caused the maternal neglect phenotype by blocking neuroplastic changes during pregnancy from occurring rather than because LHb function is actually necessary to ongoing maternal behavior in the parturient mouse dam. To compare the effect of LHb inactivation on novel maternal behaviors and established behaviors in primiparous dams, we examined pup retrieval (after scattering by the experimenter) and nest building (Figs. 3, 4, Extended Data Fig. 3-1, Movies 3 and 4) and analyzed with a random intercept linear mixed model (Fig. 5; Extended Data Fig. 5-1). DREADD-treated dams failed to learn to retrieve their pups across MBTs performed on LD1–LD4. The control-treated dams learned to retrieve their own pups over the same period. Simultaneously, nesting behavior was suppressed in DREADD-treated dams compared with control dams.

Finally, we conducted a spatial analysis comparing remaining NeuN+ LHb neuron counts in kainic acid-treated and saline-treated dams along the A–P axis. As expected, saline-treated dams have fewer neurons in the smaller anterior LHb than in the larger, posterior LHb (Fig. 7). We found that remaining NeuN+ neuron counts in the posterior element of LHb were most predictive of maternal behavior outcomes (Fig. 7; Extended Data Figs. 7-1 and 7-2). The animals included in our analysis had central- and posterior-biased LHb lesions (Fig. 7; Extended was Fig. 7-1), so this leaves open the possibility a larger sample of anterior-restricted LHb lesions could produce similar results. Our sole data point of an anterior-restricted lesion suggests anterior-restricted LHb lesions do not impact maternal behavior, but our findings on this point remain inconclusive due to lack of sample size (Extended Data Fig. 7-2). So, future experiments would benefit from specific targeting of anterior versus posterior LHb.

Random intercept linear mixed models were utilized because in both the chronic chemogenetic inactivation experiments and the kainic acid spatial histology data, the data were hierarchical in nature (each animal contributed multiple data points). The linear mixed models enable the multivariate analysis of hierarchical data (in this case, across time in Fig. 5 and across space in Fig. 7; for more see Results section or Verbeke, 1997; Barr, 2013; Bates et al., 2015).

Here, we establish that the LHb is required for maternal behavior in the naturally parturient mouse dam. Work from the Morrell Lab in the 1990s examined the role of the LHb in maternal behavior in the rat dam (Matthews-Felton et al., 1995), but the findings have yet to be extended to the mouse dam nor the mouse dam studied in conjunction with behavior toward her own biological pups. Virgin female mice are not innately pup-avoidant like virgin female rats and through sensitization exhibit a similar array of parental behaviors to the parturient mouse dam (Leblond, 1938; Kohl and Dulac, 2018). This leads sensitized virgin female mice to frequently be used, instead of lactating dams, to study mouse parenting behavior. However, virgin mouse females’ parental behavior acquisition, hormonal environment, gene expression, and motivational state all differ significantly from those of naturally parturient dams (Gandelman et al., 1970; Hauser and Gandelman, 1985; Abellán-Álvaro et al., 2021; Carcea et al., 2021). By establishing the role of the LHb in maternal behavior in the naturally parturient mouse dam, we solidify the LHb as a component of shared parental circuitry between alloparental virgin females and naturally parturient mouse dams.

One alternative explanation of our findings would be an adjacent off-target area incidentally lesioned with kainic acid or infected with DREADD virus being responsible for the maternal neglect phenotype. We find this possibility unlikely, since kainic acid-lesioned animals’ neighboring brain regions were scored for NeuN+ cell loss (see Materials and Methods) and all kainic acid-lesioned animals received total scores of 0, for no apparent cell loss in any of the kainic acid-lesioned animals’ neighboring brain regions, and thus no further analysis was conducted. In the chemogenetic inactivation experiments, off-target transgene expression was fairly common. Off-target infections were particularly prevalent in the hippocampus, which was located along the injection trajectory for LHb, and in the anterior pretectal nucleus, which was highly sensitive to infection by trace amounts of virus. We verified there was no relationship between any off-target transgene expression and maternal behavior outcomes by conducting blinded semiquantitative scoring of mCherry+ cells in off-target regions and analyzing the results in relation to retrieval and nesting behavior in DREADD-treated dams. None of the analyses of off-target transgene expression versus maternal behavior approached significance (Extended Data Fig. 6-2).

Together with the decrease in c-Fos+ cells in the DREADD group compared with that in the control group (Extended Data Fig. 6-3), as well as the spatial histology data from the kainic acid lesions (Fig. 7; Extended Data Fig. 7-1), we are confident the effect of maternal behavior from these manipulations arises from perturbations of the LHb.

Kainic acid is a potent ionotropic glutamate receptor agonist that induces excitotoxicity in neurons expressing kainite and α-amino-3-hydroxy-5-methyl-4-isoxazolepropionic acid receptors. This glutamate receptor overactivation causes a massive calcium influx which stresses endoplasmic reticular membranes and mitochondrial function, eventually leading to neuronal death via apoptosis and necrosis (Schinder et al., 1996; Shacka et al., 2006; Dong et al., 2009; Mattson, 2017). The LHb has been established as a brain region susceptible to kainic acid-induced excitotoxicity in the C57BL/6 mouse following systemic kainic acid administration (Benkovic et al., 2006). Kainic acid kills neurons, spares fibers of passage, and, directly or indirectly, leads to astrocyte and microglial activation as evidenced by reactive gliosis following kainic acid administration (Chen et al., 2005; Benkovic et al., 2006; Dong et al., 2009; Zhang and Zhu, 2011).

AAVs driven by the human synapsin (hSyn) promoter enable neuronally restricted transgene expression (Kügler et al., 2003; Haenraets et al., 2017; Radhiyanti et al., 2021). The chemogenetic receptor hM4Di, when ligand-bound, activates G-protein inward–rectifying potassium channels, thereby hyperpolarizing the cell membrane and silencing neural activity (Armbruster et al., 2007). Ligand-bound hM4Di has been shown to inhibit neural activity in the LHb (Extended Data Fig. 6-3; Nair et al., 2012). Injecting saline or AAV into the brain causes limited reactive gliosis of astrocytes and microglia at the injection site (McLeod et al., 2024). Thus, the two experimental treatment groups, kainic acid LHb-injected animals and AAV-hSyn-hm4di-mCherry LHb–injected animals, both underwent a pan-neuronal manipulation (ablation or inactivation, respectively), accompanied by increased astrocyte and microglial activity at the injection sites.

During DREADD surgeries, we had no notion as to a particular zone of interest along the A–P axis of LHb, so we injected each animal with multiple viral injections along the A–P axis in an attempt to ensure anterior, central, and posterior LHb were all infected. Future work could use single bilateral viral injections targeted to posterior LHb to greatly limit off-target infections. The experimental design of the chemogenetic inactivation experiments did not enable spatial histology analysis since dividing the DREADD-treated dams into maternal behavior outcome-based groups was nontrivial given the heterogeneity of some dams’ maternal behavior (such as dams who engaged in retrieval but not nesting or the reverse; Fig. 5).

The severe maternal neglect phenotype following kainic acid lesion and the maternal behavior deficits caused by chronic LHb inactivation are likely related to reward signaling in the regulation of maternal behavior. Maternal behavior, like all motivated behavior, requires tracking “value changes,” e.g., “a pup has just fallen out of the nest,” and “value states,” e.g., “that pup's distress call is aversive,” to motivate flexible decision-making, e.g., a dam retrieving the displaced pup to the nest (Proulx et al., 2014). Through input from the ventral pallidum and reciprocal connectivity with the serotonin and dopamine systems, the LHb plays an important role in the integration of value changes and value states (Reisine et al., 1982; Amat et al., 2001; Matsumoto and Hikosaka, 2007; L-M. Yang et al., 2008; Kim, 2009; Omelchenko et al., 2009; Bromberg-Martin and Hikosaka, 2011; Stamatakis and Stuber, 2012; Jhou et al., 2013; Stamatakis et al., 2013; Amo et al., 2014; Brown et al., 2017; Stephenson-Jones et al., 2020; Lalive et al., 2022; Mondoloni et al., 2022).

There is strong evidence of spatial segregation of LHb cell types. Geisler et al. (2003) conducted immunohistochemical analysis of 30 antibody markers in rat habenular sections and found specific subnuclei marked by neurofilament H, Kir3.2 protein, GABA B receptor, and tyrosine hydroxylase (axonal). Quina et al. (2015) performed extensive LHb-efferent tracing in the mouse, using *Pou4f1* antibodies to label the posterior two-thirds of LHb and finding retrograde median raphe injections preferentially label the medial division of LHb. Mitra et al. (2003) and Milner et al. (2010) both show significant estrogen receptor beta expression in the posterior third of LHb. Two single-cell RNA–sequencing and in situ hybridization of the mouse habenula projects were undertaken by two separate labs (Hashikawa et al., 2020; Wallace et al., 2020) and showed substantial agreement, including large populations of cells with transcripts for *Pcdh10*, *Gpr151*, *Chrm3*, and *Gap43*, all of which are spatially divided. *Gap43* shows topographic overlap with the neurons of the medial division that Quina et al. (2015) found to project to median raphe. VTA-dopamine neurons seem to preferentially receive input from *Chrm3*+ LHb cells (Wallace et al., 2020). *Necab1* and *Cartpt* represent still other spatially organized cell populations in LHb (Hashikawa et al., 2020). Thus, future work could conduct in vivo recordings of LHb cells with specific genetic markers or projection targets, such as *Gap43* or the median raphe, which seem to largely receive input from neurons along the medial division of LHb and record neural activity during maternal behavior. This would allow analysis of differential involvement of a given neuronal subpopulation in the established nest building behavior versus novel maternal behaviors of pup retrieval and crouching behaviors. This could build on prior findings in deer mice that nest building may be largely genetically independent from pup retrieval and crouching which may be largely regulated together by genes at the same loci (Bendesky et al., 2017). In the same animals, neural responses to perturbations of value changes and value states could be assessed, enabling significant progress toward understanding the transformation of pup-related stimuli into maternal behavior in the mouse dam.

Given our findings that the LHb is required for maternal behavior in the naturally parturient mouse dam and the wealth of evidence that the LHb, as a critical regulator of reward signaling, is also involved in the pathophysiology of depression and anxiety (Matsumoto and Hikosaka, 2007; Sartorius and Henn, 2007; L-M. Yang et al., 2008; B. Li et al., 2011; Proulx et al., 2014; Shabel et al., 2014; Y. Yang et al., 2018), we believe there is substantial evidence to warrant the investigation of the LHb as a possible target for future therapeutics in the treatment of postpartum depression and anxiety (M. Li, 2022).

## Synthesis

Reviewing Editor: Michael Michaelides, NIDA-NIH Decisions are customarily a result of the Reviewing Editor and the peer reviewers coming together and discussing their recommendations until a consensus is reached. When revisions are invited, a fact-based synthesis statement explaining their decision and outlining what is needed to prepare a revision will be listed below. The following reviewer(s) agreed to reveal their identity: Fereshteh Nugent, Hiroyuki Ichijo.

Reviewer 1

In this interesting study, the authors used kainic acid lesions within the LHb and chronic Gi-DREADD chemogenetic silencing of LHb neurons to show the involvement of LHb in maternal behavior in the naturally parturient primiparous mouse dam where they demonstrate interesting data for a significant loos/reduction in maternal care with LHb lesion as well as chemogenetic inhibition. They also provided some anatomical data for the possibility of the importance of posterior LHb in maternal behavior in mice. Overall, this study provides additional evidence for the role of LHb function in maternal behavior in mice that is less understood. Some of the most interesting aspect of their findings is the involvement of the LHb in learning of pup retrieval behavior as a novel maternal behavior in primiparous mouse dams. The use of multivariate multi-level random intercepts model is also an excellent approach for their rigorous analysis of the data. Overall, the experiments are appropriately designed and technically sound and the data are clearly presented and most conclusions are supported by the data, however additional experiments/explanations should be included to further validate and clarify some of the claims/interpretations. Here are several major and minor issues that need to be addressed.

1. There is no data showing the success of DREADD approach in silencing LHb neurons. Is there any physiological recording or c-fos experiments to confirm that Agonist 21 indeed decreased LHb neuronal activity in Gi-DREADD expressing LHb neurons? This verification needs to be included.

2. It occurs to the reviewer that the way in which the authors subdivide the LHb into anterior and posterior regions for the purpose of reporting the number of neurons in kainic acid lesions and Gi-DREADD experiments is mostly arbitrary, as there is no morphological, molecular or functional distinction between LHb subregions in this study with the exception of the reported AP axis of LHb. Specifically, there are numerous studies that have divided the LHb into several subnuclei as also discussed in the discussion by the authors and also this paper brain for example https://doi.org/10.1038/s41598-022-14328-1

It would be interesting if in the anatomical studies, the authors perform some additional analysis of behavioral data within these subregions of LHb that are reasonably well-established for rodent.

3. The authors report in the DREADD experiments that "many of the dams with severely impaired pup retrieval had quite modest LHb transgene expression". Since there is variability in the spread of drug/virus within the LHb and heterogeneity of LHb, again it would be more convincing to have carried out DREADD based c-fos studies in LHb output regions, or have expressed the DREADD in a cfos inducible manner to show in which subregion of LHb these cells reside and are important for mediating maternal behaviors. IHC analysis of mcherry+DREADD neurons is commonly used as a confirmation for the success of targeting the LHb in chemogenetics but without any functional correlation for the DREADD approach, counting the mCherry-labeled neurons in the anatomical study is less convicting.

Reviewer 2

This study investigated the contribution of the LHb to the establishment of maternal behaviors. The authors analyzed a series of behaviors in the maternal behaviors, by utilizing cell ablation and chronic functional inhibition, to temporally separate the learning. Furthermore, the authors suggested functional topographic differences between the anterior and posterior LHb in the establishment of maternal behaviors by using the statistical approach, which is novel. This reviewer suggests that the following improvements would enhance the value of this paper.

(1) The experiments comparing the effect of kainic acid on cell death with the effect of DREADDs on learning inhibition were important. The administration of kainic acid intended to cause neuronal cell death due to excitotoxicity. Is the target of kainic acid the same as the target of DREADDs using hSyn-hM4D(Gi)? Or are they different? And to what extent are they the same and to what extent are they different? If these can be confirmed experimentally, that would be the best. Or can it be discussed with proper citation of previous studies? Based on the above, to what extent can the authors argue that the mechanisms of the effects of kainic acid and DREADDs are the same? This reviewer thought that the authors are able to properly convey the value of this study to the readers If the authors can organize and discuss these knowledge and experimental results. The above should be considered.

(2) In the spatial histology random intercepts model, the brain coordinates of the LHb should be shown when the LHb is divided into 10 sections along the AP axis. In addition, histological images of typical LHb segments are helpful for the reader's understanding. As shown in Figure 7, the anterior part of the mouse LHb is generally smaller than the posterior part, resulting in fewer cells. In addition, the posterior LHb is composed of more diverse subnuclei than the anterior, and therefore, this structure is expected to have diverse functions. This should be included when interpreting the results of the kainic acid experiment in random interceps.

(3) The author mentioned in the abstract and the beginning of the introduction that the importance of the maternal behaviors can be talked about in the context of evolution. The evolution-related argument described in this paper is plausible; however, the results and discussion showed no evolution. It is often easy to fall into a general tendency to go to excess when discussing evolution, partly because it is difficult to describe evolution empirically. The introduction about the evolution here would also be a bit over-speculation; it should be rewritten. Because the final statement in the discussion ended with the possibility of clinical applications, there is an option to use a clinical introduction at the beginning of the paper.

(4) Regarding the following statement (Page 18 the last paragraph),

"kainic acid lesioned animals' neighboring brain regions were closely inspected for loss of NeuN+ neurons and none were found."

How did the authors examine the loss of NeuN+ neurons? Did the authors count them?

Minor points

(1) P7 line 22

"20x objective" probably the letter x is wrong. The correct letter is "✕".

(2) P7 line 26

About "Gpr-151 staining", it is probably "anti-GPR151 immunostaining". This is the only mention of anti-GPR151 immunostaining and there is no photo anywhere. If the photo is not necessary, is it necessary to mention this antibody?

(3) P9 line 22

(p >. 01, Wilcoxon rank sum test in all comparisons, unless otherwise stated)

Is this the correct direction for this inequality? There are many similar places (8 in the text and 1 in Fig. 2 legend), so please check all of them and correct them.

(4) P10 line 4

(IQR) = 9,239 (8615.75-9998)

Is this comma correct? It does not seem to be used like this in other places. Please check.

(5) P13 the last line and P14 line 2

It is not usual for Figure 6C to appear first and then Figure 6B in the text.

(6) P10 line 12, P14, P15, P17 about "surviving NeuN+ LHb neuron".

In "surviving NeuN+ LHb neuron counts in kainic acid-treated and saline-treated dams along the anterior-posterior axis (P17)",

is "survive" the right word, especially in the case of saline-treated dams? It should be " remaining NeuN+ LHb neuron in kainic acid-treatment"?

(7) P19 line 9

I think it would be better to avoid subjective wording and use objective phrases. For example, the verb "feel" should be avoided in the following, "we feel confident the effect of maternal behavior from these manipulations arises from perturbations of the LHb".
