# Supplementary figures and images for: The Lateral Habenula Is Necessary for Maternal Behavior in the Naturally Parturient Primiparous Mouse Dam
Source: eNeuro. 2025 Jan 8;12(1):ENEURO.0092-24.2024. doi: 10.1523/ENEURO.0092-24.2024 (PMC11734883; doi:10.1523/ENEURO.0092-24.2024)

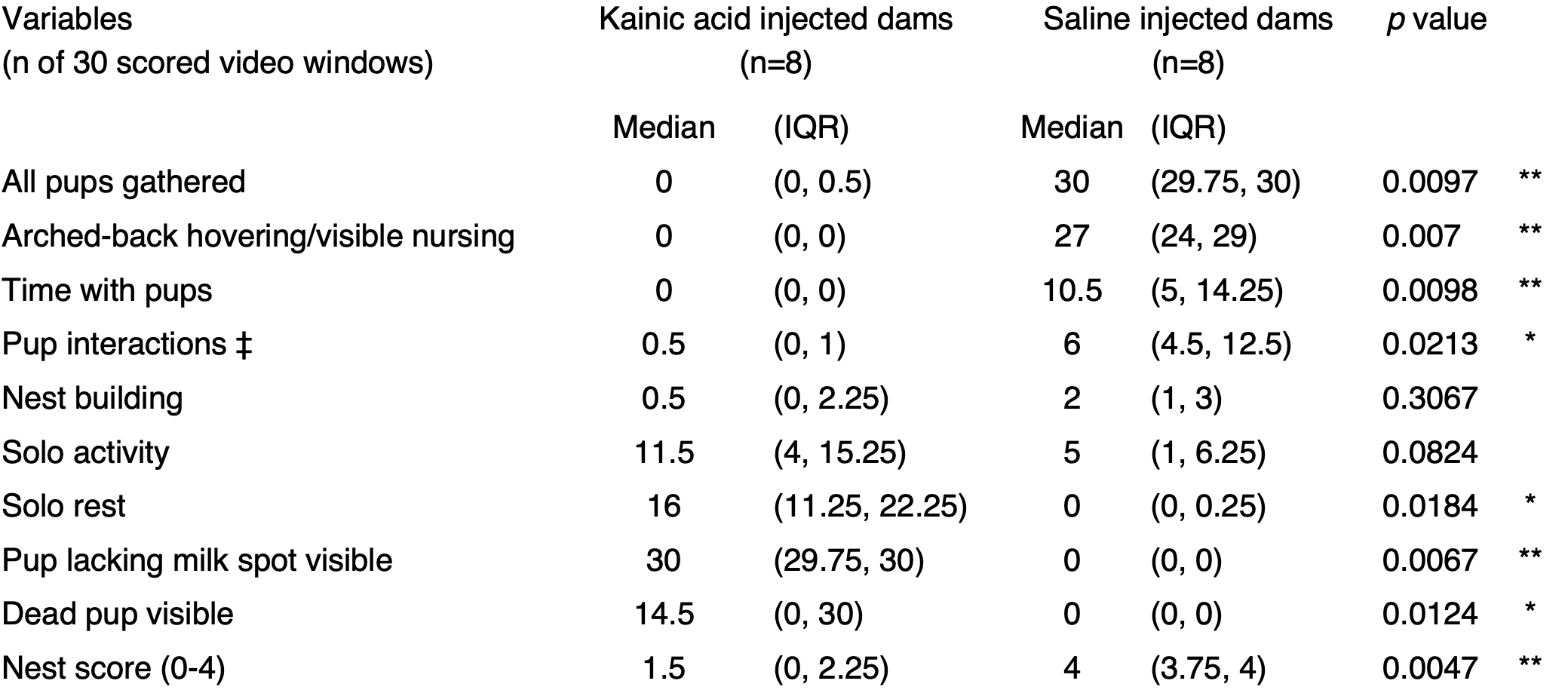

Supplement: Figure 1-1 — Table showing all the behavioral results from the kainic acid lesion experiments. Medians and IQRs are provided by group, and p-values were calculated using the Wilcoxon rank sum test. P-values were left unadjusted for multiple comparisons in concordance with the recommendation from the Johns Hopkins Biostatistics Center. Note the difference in scale from 0-30 (variables 1-9), and variable 10 (nest score), which is on a 0-4 scale. See the Methods section for further details on the nest score scale. ‡ Pup interactions include pup sniffing, inspecting, anogenital licking, or retrieval. Download Figure 1-1, TIF file. [file eneuro-12-ENEURO.0092-24.2024-s006.tif]

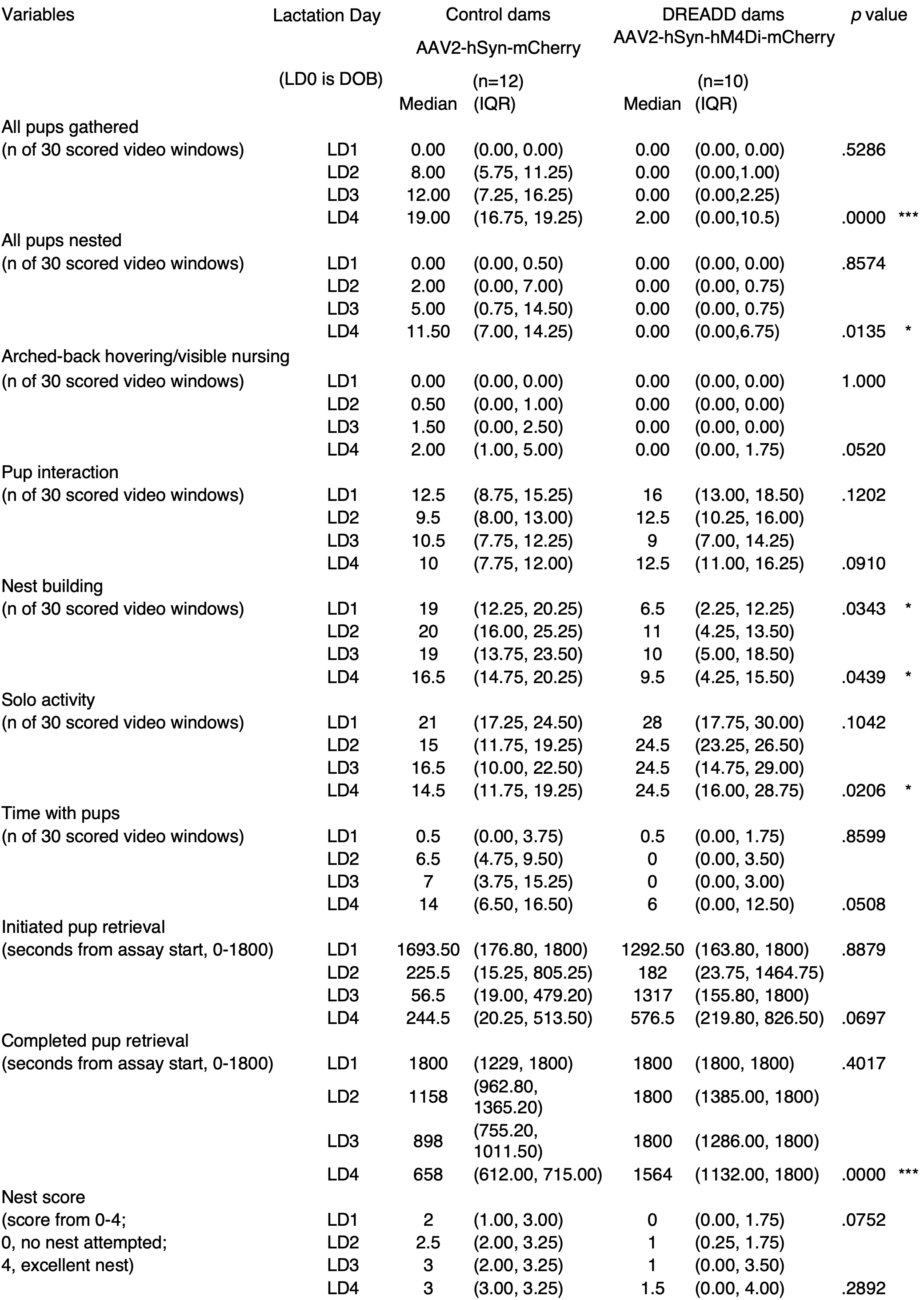

Supplement: Figure 3-1 — Table showing all the behavioral variables from chronic chemogenetic inactivation experiments. Medians and IQRs are provided by group for LD1-LD4, and p-values were obtained by the Wilcoxon rank sum test. P-values are left unadjusted for multiple comparisons, in concordance with the recommendation from the Johns Hopkins Biostatistics Center, since all comparisons were planned prior to data collection, comparisons were made to test specific hypotheses, and the number of comparisons were on the order of dozens, not thousands. Note the difference in scale from 0-30 (variables 1-9), and variables 10-12 (0-1800, 0-1800, and 0-4 scales respectively). See Methods section for further detail on the scales. Download Figure 3-1, TIF file. [file eneuro-12-ENEURO.0092-24.2024-s007.tif]

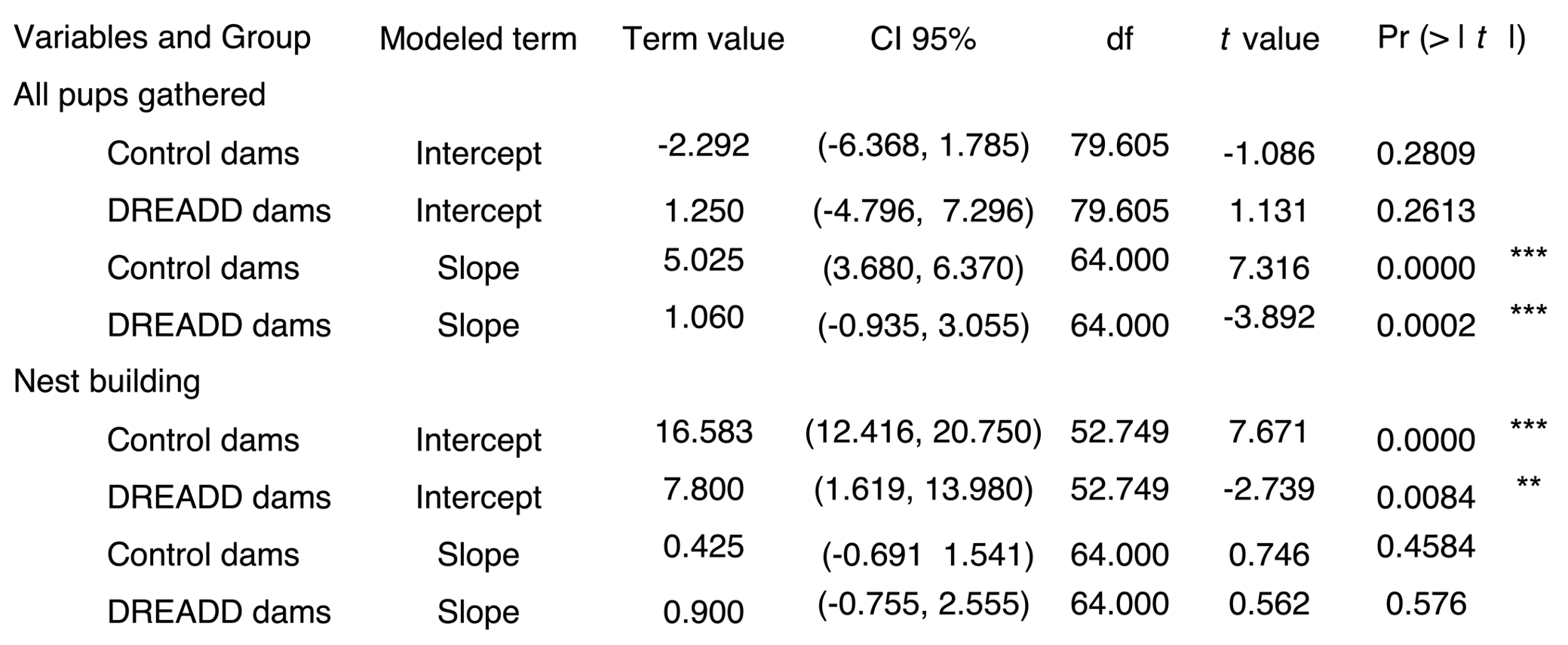

Supplement: Figure 5-1 — Table of values plotted in Figure 5. Table showing the Control and DREADD-treated group intercepts and group slopes for the random intercepts model depicted in Figure 5. For the “all pups gathered” variable, a near-zero intercept for both control and DREADD groups suggests a novel behavior on LD1, and a steep positive slope suggests rapid acquisition of the pup retrieval behavior for the control group. The much smaller slope for the DREADD group suggests their treatment impaired pup retrieval learning. For the “nest building” variable, the near-zero slopes for both groups are consistent with an established behavior. The much lower group intercept for the DREADD group suggests nest building performance was impaired by their treatment. Confidence intervals, degrees of freedom, t values, and significance testing for each term is shown in subsequent columns. Significance testing for the control group rows in this table reflect the model’s confidence that the true term is nonzero, and the significance testing for the DREADD group rows in this table reflect the model’s confidence that there are statistically significant differences between the control group and the DREADD group. Download Figure 5-1, TIF file. [file eneuro-12-ENEURO.0092-24.2024-s008.tif]

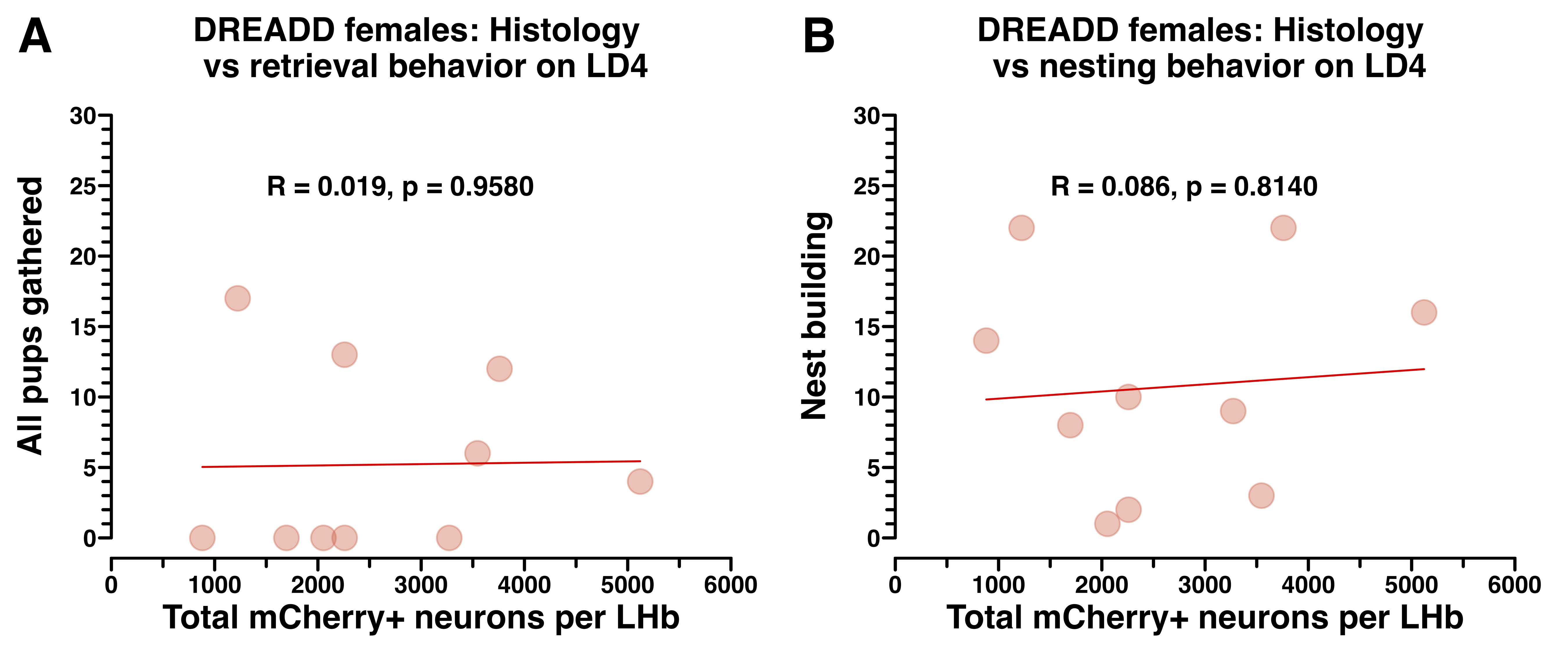

Supplement: Figure 6-1 — mCherry + LHb cell counts do not predict maternal behavior outcomes in DREADD-treated dams. A) Pearson correlation between MBT score for the “all pups gathered” variable (out of 30) and total + mCherry cells counted per LHb in DREADD-treated dams shows no correlation (R = 0.0019, p = .9580). B) Pearson correlation between MBT score for “nest building” (out of 30) and total mCherry + cells counted per LHb in DREADD-treated dams shows no correlation (R = 0.086, p = .8140). The number of mCherry + cells in the LHb does not appear to predict maternal behavior outcomes in DREADD-treated dams. Download Figure 6-1, TIF file. [file eneuro-12-ENEURO.0092-24.2024-s009.tif]

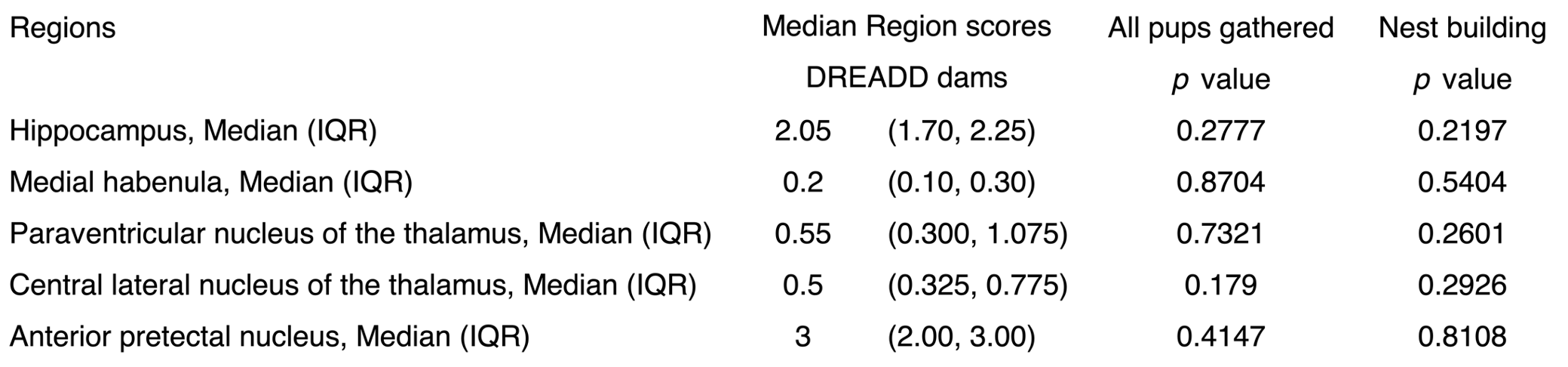

Supplement: Figure 6-2 — Off-target transgene expression in neighboring brain regions do not predict maternal behavior in DREADD group dams. The five brain regions most closely bordering LHb were subjectively blind-scored for transgene expression magnitude (-, +, ++, +++) (see methods section for rubric) on each brain section for each DREADD-treated dam. The scores, produced by a single scorer, were totaled across all sections for each dam, then a Spearman correlation was performed comparing “all pups gathered” and “nest building” scores against total transgene expression scores for each off-target region. No regions were statistically significant. Download Figure 6-2, TIF file. [file eneuro-12-ENEURO.0092-24.2024-s010.tif]

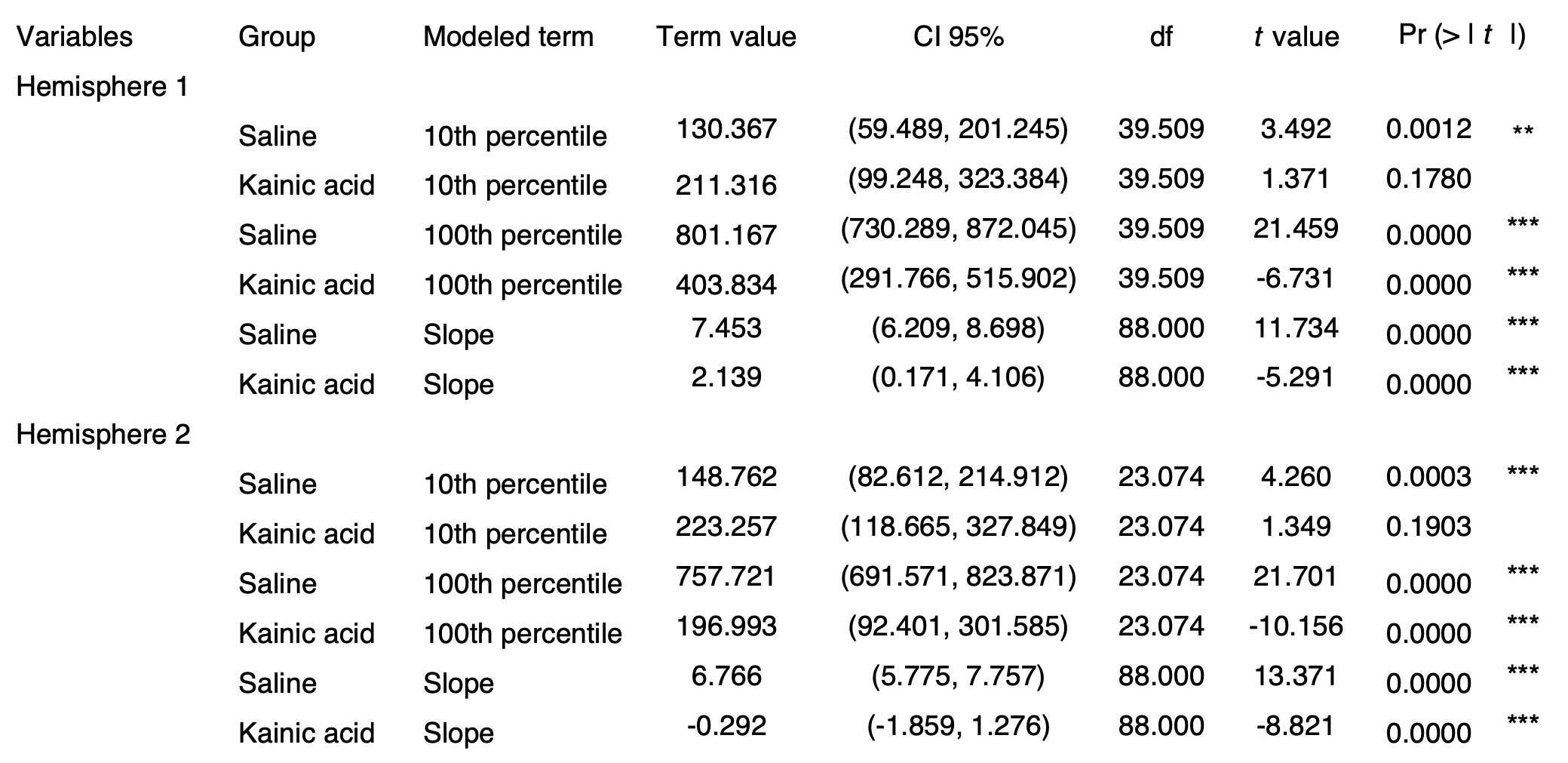

Supplement: Figure 7-1 — Table of specific term values plotted in Figure 7. The group terms “10 percentile” and “100th percentile” refer to the value of the y-value at those deciles, in this case the number of neurons remaining in the first and tenth deciles of the AP axis. Significance testing in all rows labeled “saline” represent the model’s certainty that the saline group’s true term value (10th or 100th percentile y-value, or slope) is nonzero, while the “kainic acid” rows’ statistical significance column denote the model’s certainty that there is a statistically significant difference between the saline and kainic acid groups. The model fails to find a significant difference between saline and kainic acid group y-values at the 10th percentile, while finding a statistically significant difference at the 100th percentile. This means the posterior aspect of LHb may be where the maternally relevant neurons reside. Slopes provided in the table are computed as if the first decile is the intercept, and thus a large positive group slope, like that of the saline group, means there were many neurons at the posterior aspect of LHb. The comparatively slight (Hemisphere 1) or negative (Hemisphere 2) group slope for the kainic acid group represents their relative paucity of neurons remaining in posterior LHb. Download Figure 7-1, TIF file. [file eneuro-12-ENEURO.0092-24.2024-s012.tif]

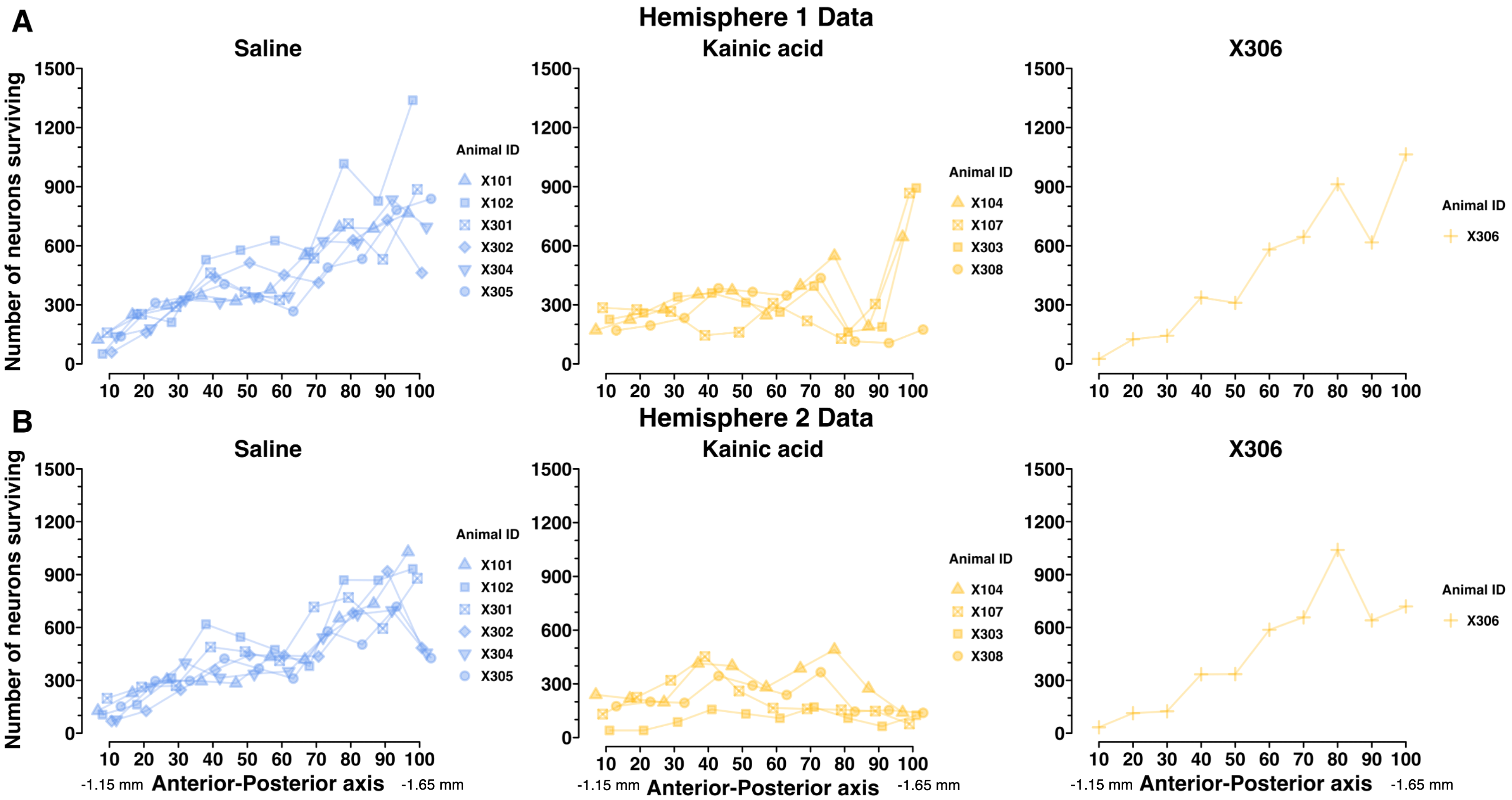

Supplement: Figure 7-2 — Spatial histology comparison of Animal X306’s remaining neuron counts across AP axis of LHb. X306 received kainic acid treatment but mothered her pups indistinguishably from the saline group, and her posterior LHb appears intact. A) NeuN + LHb neuron counts in Hemisphere 1 for each decile of the AP axis of LHb for the saline group, the kainic acid group, and animal X306 (left, middle, right, respectively). B) NeuN + LHb neuron counts in Hemisphere 2 for each decile of the AP axis of LHb for the saline group, the kainic acid group, and animal X306 (left, middle, right, respectively). Download Figure 7-2, TIF file. [file eneuro-12-ENEURO.0092-24.2024-s013.tif]
